# Comprehensive metabolomics and phytochemical analyses identified important metabolites involved in the antioxidant activity of four Swiss chard cultivars (*Beta vulgaris* L. var. cicla) with different leaf colours

**DOI:** 10.1016/j.fochx.2026.103587

**Published:** 2026-01-25

**Authors:** Chanung Park, Jae Kwang Kim, Jinsu Lim, Kihyun Kim, Haejin Kwon, Eun Sol Cho, Ye Jin Kim, Moon-Sub Lee, Sujatha Ramasamy, Ramaraj Sathasivam, Sang Un Park

**Affiliations:** a Department of Crop Science, Chungnam National University, 99 Daehak-ro, Yuseong-gu, Daejeon 34134, Republic of Korea; b Division of Life Sciences and Convergence Research Center for Insect Vectors, Incheon National University, Yeonsu-gu, Incheon 22012, Republic of Korea; c Department of Smart Agriculture Systems, Chungnam National University, 99 Daehak-ro, Yuseong-gu, Daejeon 34134, Republic of Korea; d Department of Crop Science, Chungbuk National University, 1 Chungdae-ro, Seowon-gu, Cheongju 28644, Republic of Korea; e Institute of Biological Sciences, Faculty of Science, University of Malaya, 50603 Kuala Lumpur, Malaysia; f Department of Integrative Agriculture, College of Agriculture and Veterinary Medicine, United Arab Emirates University, P.O. Box 15551, Al Ain, Abu Dhabi, United Arab Emirates

**Keywords:** *Beta vulgaris* L. var. cicla, Swiss chard, Different leaf cultivars, Primary and secondary metabolites, Metabolic profiling, Antioxidant activities

## Abstract

*Beta vulgaris* L. var. cicla is a leaf vegetable and has been used in many cuisines worldwide for a long time. The long history of Swiss chard cultivation has resulted in the development of many cultivars through domestication and hybridisation. The diversification of Swiss chard cultivars has resulted in differences in metabolite profiles. However, comparative studies analysing metabolites among cultivars are still lacking. In this study, 63 metabolites were identified in four Swiss chard cultivars with different leaf petiole colours using HPLC, GC–qMS, and GC–TOFMS. In addition, the antioxidant activity, TPC, and TFC were determined in extracts from the four Swiss chard cultivars. Comprehensive metabolic profiling analysis was performed using metabolites identified in Swiss chard. In addition to flavonoids, the metabolomic data and antioxidant assay identified non-flavonoid metabolites, such as policosanol, carotenoids, and glutamic acid derivatives, as strong antioxidant candidates that were not previously recognized as antioxidants in Swiss chard.

## Highlights

• Metabolic profiling and antioxidant activities in different Swiss chard leaves.
• Totally 63 known metabolites were identified in different leaves.
• The heat map showed that most metabolites were high in red and white cultivars.
• KEGG enrichment analysis showed that various nutraceutical pathways were impacted.
• Several non-flavonoid metabolites might responsible for the antioxidant activity.

## 1 Introduction

*Beta vulgaris* L. var. cicla (*B. vulgaris*) is an economically important vegetable that is extensively used worldwide. Although the origin and evolutionary history of *B. vulgaris* are not clear, the long history of hybridisation and domestication since 8500 BCE has resulted in many cultivars (Galewski & McGrath, 2020). *B. vulgaris* is generally classified into four groups, namely chard, sugar beet, fodder beet, and table beet, depending on their phenotypes (Galewski & McGrath, 2020). Unlike other *B. vulgaris* varieties, chard, commonly known as Swiss chard, does not have enlarged taproots and is used as a leaf vegetable. It is also known to be the most genetically diverse among the groups, which may be the result of its earliest domestication (Galewski & McGrath, 2020). This diversity is reflected by the colouration observed in Swiss chard leaves depending on the cultivar. These phenotypic differences in colour among the cultivars suggest variations in the content of secondary metabolites, such as carotenoids, betalain, and other phenolics that are nutritionally beneficial to the human diet.

Metabolites are compounds in plants that play roles in plant survival and have many beneficial biological effects when consumed by humans. Metabolites can be classified into two groups and are generally classified as primary metabolites when they are essential for the organism to maintain its function; these include amino acids, carbohydrates, lipids, and nucleic acids (Kumar, Asthana, Gupta, Nigam, & Mahajan, 2018). Secondary metabolites, such as phenylpropanoids, anthocyanins, and carotenoids, are metabolites that have a relatively indirect role in plant growth and development; they have roles in pollination and defence against stress (Sathasivam et al., 2025). Secondary metabolites are in the spotlight because of their potential biological activity, which can be used in many fields, such as agriculture, food, cosmetics, and pharmaceuticals (Selwal et al., 2023). These metabolites have recently been comprehensively and simultaneously analysed using metabolomics, while traditional metabolite studies target a small number of metabolites and certain types (Clish, 2015). Thus, metabolomics tends to provide more ideas for understanding the relationships among metabolites, their pathways, and metabolic profiles in the organism.

Previously, studies have investigated the contents of secondary metabolites, including betalain, carotenoids, and phenolic compounds, in Swiss chard (Barickman & Kopsell, 2016; Lisiewska, Kmiecik, & Korus, 2006; Montoya-Arroyo et al., 2022). In addition, the antioxidant activity of Swiss chard has been reported in previous research. An antioxidant study comparing the leaves and the stems of Swiss chard has shown that both tissues have an antioxidant effect, although the leaves have a higher content than the stems (Pyo, Lee, Logendra, & Rosen, 2004). In addition, a comparative study between Swiss chard and beetroot leaves has shown that the antioxidant activity of Swiss chard extract may be attributed to phenolic acid (Zein, Hashish, & Ismaiel, 2015). A study on the flavonoid fraction from Swiss chard also shows that flavonoids are responsible for antioxidants (Ninfali et al., 2007). Previous studies have also investigated the secondary metabolites and antioxidant activity of Swiss chard. However, no studies have comparatively analysed multiple metabolites among four Swiss chard cultivars at the metabolomic level. Only a few studies have explored the lipophilic metabolites, including policosanol, among Swiss chard cultivars (Almeida et al., 2025).

Thus, in this study metabolites were analysed with three Korean local Swiss chard cultivars, ‘Back Gyeong’, ‘Cheong Gyeong’, and ‘Hwang Gyeong’, which have white, green, and yellow petioles, respectively, are common in the Korean groceries and seed markets, and one global cultivated cultivar called ‘Ruby Red’, which has red petioles. In detail, primary and secondary metabolites, including betacyanin, betaxanthin, phenylpropanoid, carotenoid, hydrophilic, and lipophilic compounds, were identified among the four Swiss chard cultivars with white, green, yellow, and red leaf petioles using High Performance Liquid Chromatography (HPLC), gas chromatography–quadrupole mass spectrometry (GC–qMS), and gas chromatography–time-of-flight mass spectrometry (GC–TOFMS). Additionally, the antioxidant activity was investigated in four Swiss chard extracts. Lastly, a comprehensive analysis was performed among the identified metabolites, antioxidant activity, and cultivars. This study will provide metabolite profiles showing the biological activity of four Swiss chard cultivars, offering knowledge of the metabolites benefiting Swiss chard consumption.

## 2 Materials and methods

### 2.1 Plant materials

Seeds of *B. vulgaris* ‘Back Gyeong’ (hereafter white cultivar), ‘Hwang Gyeong’ (hereafter yellow cultivar), ‘Cheong Gyeong’ (hereafter green cultivar), and ‘Ruby Red’ (hereafter red cultivar) chard cultivars were purchased from Asia Seed Co., Ltd. (Seoul, Korea). Four chard cultivars were grown in a greenhouse at Chungnam National University, Daejeon, Korea, between April and June, with the temperature maintained between 25 °C–27 °C with 16 h of light and 8 h of dark. The chard leaves were harvested after 40 days (**Fig. S1**). The samples were then freeze-dried at −50 °C for at least 72 h and ground into a fine powder using a mortar and pestle for phytochemical analysis. All metabolomic and gene expression analyses were performed using three biological replicates (*n* = 3), each analysed in triplicate.

### 2.2 Carotenoid extraction and quantification

Carotenoid extraction was performed as described in a previous study with some modifications (Sathasivam et al., 2025). Freeze-dried powders of four Swiss chard cultivar leaves were prepared as mentioned above, and 3 mL of ethanol containing 0.1% ascorbic acid was added to 10 mg of the powder. Samples were vortexed and incubated at 85 °C for 15 min, and then 120 μL of 80% potassium hydroxide solution was added. The samples were incubated on ice for 5 min, and the following were added: 1.5 mL hexane, 1.5 mL ddH_2_O, and 50 μL β-apo-8′-carotenal as an internal standard (IS). Samples were centrifuged at 14,000 ×*g* for 10 min at 4 °C, and the top layer (hexane layer) was transferred to new tubes. Additional extraction was performed by adding 1.5 mL of hexane to the bottom layer. The hexane layer was dried using a vacuum concentrator and restored with 250 μL of solvent composed of methanol and dichloromethane (1:1, *v/v*). The restored samples were analysed using high-performance liquid chromatography (HPLC), following the method described by Sathasivam et al. (2025).

### 2.3 Quantification of betalain contents

Betalain quantification was executed as described in a previous study (Cano, Gómez-Maqueo, García-Cayuela, & Welti-Chanes, 2017). A mixture containing 100 mg powdered Swiss chard leaves and 2 mL of distilled water was sonicated, and the supernatant of the mixture was isolated using a centrifuge at 12000 rpm for 15 min at 4 °C. The absorbances of the ten-time diluted extracts to water were measured at 535 nm for betacyanin and 483 nm for betaxanthin using a spectrophotometer (SPECTROstar Nano microplate reader, BMG LABTECH). Values attained from the spectrophotometer were used to quantify the betacyanin and betaxanthin using the equation of Cano et al. (2017).

### 2.4 GC-qMS analysis of lipophilic compounds

Lipophilic compounds (tocopherol, tocotrienols, phytosterols, and policosanols) from four Swiss chard cultivars were analysed by using GC-qMS as described previously (Sathasivam et al., 2025). In a 15-mL tube, 10 mg of freeze-dried sample was extracted in 3 mL of 0.1% ascorbic acid in ethanol (w:v) and 0.05 mL of 5α-cholestane (10 mg/mL) as an internal standard (IS) in a 15-mL tube. The samples were placed in a water bath at 85 °C for 5 min for extraction, followed by mixing with 120 μL of 80% potassium hydroxide (w:v) to saponify the sample. The samples were placed in a water bath at 85 °C for 10 min after vortexing. The samples were then incubated on ice for 5 min, and 1.5 mL of hexane and 1.5 mL of deionised water (DW) were added. After vortexing, the mixture was centrifuged at 1200*g* for 5 min at 4 °C. The upper layer was transferred to a 15-mL tube, and the pellet was mixed with 1.5 mL of hexane for re-extraction. After vortexing, the mixture containing the pellet was centrifuged, and the upper layer was transferred once more. The sample was dried under nitrogen gas until it reached 1 mL and evaporated in a vacuum centrifugal concentrator (VS-802F, Visionbionex, Gyeonggi, Korea) for 1 h. After adding 0.03 mL *N*-methyl-N-(trimethylsilyl)trifluoroacetamide (MSTFA) and 0.03 mL pyridine to the mixture to derivatise the sample, the reaction was initiated using a compact thermomixer (model 5355, Eppendorf AG, Hamburg, Germany) at 30 °C and 1200 rpm for 90 min. The derivatised sample was injected in a GCMS-QP2010 Ultra system equipped with an autosampler AOC-20i (both Shimadzu, Kyoto, Japan) prepared with a Rtx-5MS column (0.25 mm diameter and 0.25 μm thickness, 30 m length; Agilent, Palo Alto, CA, USA) for GC–MS. The injection temperature, interface, and ion source were 290, 280, and 230 °C, respectively. Helium gas at a 1.0 mL/min flow rate was used as a carrier. The GC programme was set at 150 °C for 2 min, followed by an increase up to 320 °C, with a ramping rate of 15 °C/min and a hold time of 10 min. The injection volume was 1 μL, and the split ratio was 1:0.1. The scaled mass range was 85–600 *m*/*z*. By comparing their retention times and mass spectra with standard compounds, Labsolutions GC–MS software version 4.11 (Shimadzu, Kyoto, Japan) was used to identify the metabolites. Absolute quantification was determined using calibration curves of each compound.

### 2.5 GC-TOFMS analysis of hydrophilic compounds

Hydrophilic compounds (amino acids, organic acids, sugars, and sugar alcohols) from four Swiss chard cultivars were obtained using GC-TOF-MS based on the previous experiment (Sathasivam et al., 2025). In a 2-mL tube, 10 mg of freeze-dried sample was extracted in 1 mL of methanol:water:chloroform = (2.5:1:1, v:v:v) solution and 0.06 mL of ribitol (0.2 mg/mL) as an IS. After vortexing, the sample was extracted using a compact thermomixer (model 5355, Eppendorf AG, Hamburg, Germany) at 37 °C and 1200 rpm for 30 min. After centrifuging the mixture at 4 °C and 16,000 ×g for 5 min, 0.8 mL of the upper layer was transferred to a 2-mL tube and mixed with 0.4 mL of DW. After vortexing, the mixture was centrifuged again, and 0.9 mL of the upper layer was transferred to a 2-mL tube. The supernatant was evaporated in a vacuum centrifugal concentrator (VS-802F, Visionbionex, Gyeonggi, Korea) for 3 h and then lyophilised using a freeze-dryer (MCFD8512, Ilshin, Gyeonggi-do, South Korea) for 16 h. After adding 0.08 mL of methoxylamine hydrochloride (MOX, 20 g/L) to the lyophilised mixture to derivatise the sample, the mixture was reacted at 30 °C and 1200 rpm for 90 min. The mixture was then added to 0.08 mL of MSTFA and reacted at 37 °C and 1200 rpm for 30 min. The derivatised sample was injected into an Agilent 7890B GC system (Agilent, Santa Clara, CA, USA) equipped with a CP-Sil 8 CB Low Bleed/MS column (30 m × 0.25 mm × 0.25 mm; CP 5860, Agilent) and a LECO Pegasus BT TOF mass spectrometer (LECO, St. Joseph, MI, USA) for GC-TOFMS analysis. The column temperature was maintained at 80 °C for 2 min, raised to 320 °C at a rate of 15 °C/min, and held at 320 °C for 10 min. The temperatures of the front inlet, transfer line, and ion source were set at 230, 280, and 250 °C, respectively. The carrier gas was helium gas at a flow rate of 1 mL/min. The injection volume was 1 μL, with a split ratio of 1:25, and the scanned mass range was 85–600 *m*/*z*. By comparing their retention times and mass spectra with standard compounds, ChromaTOF software (version 5.50; LECO) was used to identify the metabolites in the in-house and MS libraries (Nist and Wiley9). Quantification was determined by the relative ratio of the analyte peak area to the IS peak area.

### 2.6 Gene expression analysis using qRT-PCR

Total RNA was extracted using TRI reagent (MRC, Cincinnati, OH, USA) following the manufacturer's instructions. The extracted RNA quality was confirmed using NanoDrop (GE HealthCare Life Sciences, Chicago, IL, USA) and by running the sample on an agarose gel. A ReverTra Ace-α-kit (Toyobo Co. Ltd., Osaka, Japan) was used to synthesise cDNA from the total RNA. Quantitative RT-PCR (qRT-PCR) was done using 2× Real-Time PCR Master Mix (Including SFC green® I) (BioFACT, Korea), as instructed by the manufacturer. BvActin was used as a housekeeping gene to calculate the relative gene expression of individual genes, and ΔCt method was used to analyze relative gene expression. The primer information used to perform qRT-PCR is shown in **Table S1**.

### 2.7 Quantification of total phenolic content (TPC) and total flavonoid content (TFC)

The TPC and TFC in the Swiss chard samples were quantified, as described in a previous study (Lim et al., 2024). The extract was prepared by mixing the supernatant of 100 mg of sonicated, freeze-dried Swiss chard powder in 70% methanol. The extracts were diluted 50 times in 70% methanol, and 0.1 mL of the diluted extract was mixed with 0.5 mL of 2 N Folin and Ciocalteu's phenol reagent (Junsei, Yongin, Korea) and incubated for 3 min at room temperature. Subsequently, 4 mL of 20% sodium carbonate was added to the previous sample mixture and incubated in the dark for 90 min. The absorbance was measured at 760 nm using a spectrophotometer (SPECTROstar Nano microplate reader, BMG LABTECH), and an equivalent calibration curve of gallic acid (ranging from 0 to 493.75 mg/L; y = 0.0013× + 0.0505, R^2^ = 0.9933) was used to quantify the TPC.

For TFC quantification, a mixture composed of 0.5 mL of the diluted extract, 2 mL of water, and 0.15 mL of 5% sodium nitrite was prepared and incubated in the dark for 5 min at room temperature. After incubation, 0.15 mL of 10% aluminium chloride was added and incubated in the dark for 15 min. The absorbance of the final mixture was measured at 415 nm using a spectrophotometer. The equivalent calibration curve of quercetin (ranging from 0 to 500 mg/L; y = 0.002× + 0.0839, R^2^ = 0.9996) was used to quantify the TFC.

### 2.8 Antioxidant activity assay

In this study, 1-(2, 6-dimethylphenoxy)-2-(3, 4-dimethoxyphenylethylamino)) propane hydrochloride (DPPH), 2,2′-azino-bis (3-ethylbenzothiazoline-6-sulfonic acid) (ABTS), and reducing power (RP) assays were performed to investigate the antioxidant activity using a previously described method (Lim et al., 2024). Several concentrations (31.25, 62.5, 125, 250, 500, 1000 μg/mL) of the extracts were prepared by serially diluting the extracts prepared for TPC and TFC analysis. The diluted samples were placed on a 96-well plate containing 100 μL of 0.2 mM DPPH solution, followed by 30 min of incubation in the dark. The absorbances of the samples were measured at 517 nm using a spectrophotometer. The scavenging activity was calculated using the collected absorbance data and the equation suggested by Lim et al. (2024).

In the case of the ABTS assay, 150 μL of ABTS solution (7 mM ABTS in 2.5 mM potassium persulfate solution) incubated in the dark for 16 h was added to the serially diluted samples in the 96-well plates, similar to the DPPH assay. The absorbances of the samples were measured at 734 nm using a spectrophotometer. The formula that was used to calculate the scavenging activity in the DPPH assay was used to calculate the ABTS scavenging activity.

For the Reducing Power (RP) assay, serially diluted samples were prepared as was done in the DPPH and ABTS assays, and 300 μL of the sample was mixed with 300 μL of 0.2 M phosphate buffer (pH 6.6) and 300 μL of 1% potassium ferricyanide solution. The samples were incubated at 50 °C for 20 min, and 300 μL of 10% trichloroacetic acid (TCA) was added and centrifuged at 10,000 rpm for 10 min. Subsequently, 500 μL of the supernatant was transferred to a new tube. The collected supernatant was mixed with 500 μL of ultrapure water and 100 μL of 0.1% ferric chloride. The absorbance of the samples was measured at 700 nm, and a higher absorbance value indicated a greater reducing power.

### 2.9 Statistical analyses

The significances of data were determined by the *t*-test or one-way analysis of variance (ANOVA), followed by Tukey's honestly significant difference (HSD) with *p* < 0.05 using SPSS. Principal Component Analysis (PCA), heat map, Variable Importance in Projection (VIP), pathway impact, partial least squares discriminant analysis (PLS-DA), and Pearson's correlation between the *B. vulgaris* cultivar leaves were performed using MetaboAnalyst 6.0 (https://www.metaboanalyst.ca), and plots were drawn using R Studio. PCA and PLS-DA were performed using Metaboanalyst 6.0, with sample normalization and data transformation set to ‘none’, and auto-scaling was selected for data scaling. PCA was visualized by score and loading plot; the result reports the percentage of variance for PC1 and PC2 from the axis labels and does not draw inferential conclusions from PCA. The PLS-DA model was fine-tuned using cross-validation metrics, including R^2^, Q^2^, and CV accuracy. Model robustness was evaluated using a label-permutation test (*n* = 1000). Variable importance was summarized by VIP analysis. All plots and their numerical values are provided in **Table S5** and **Figs. S4**, and **S5**. Metabolites that were identified from Swiss chard, showing significant differences among the different cultivars, were annotated with Kyoto Encyclopedia of Genes and Genomes (KEGG) compound identifiers and mapped onto KEGG pathways using *Arabidopsis thaliana* as the model organism. Pathway impact values were attained from MetaboAnalyst's pathway topology analysis, where compound node significance was computed by relative betweenness centrality; for every pathway, the impact score was calculated as the total of the significance measures of the matched compounds divided by the total of the significance measures of all compounds in that pathway. PathVisio 3.3.0 was used to plot the pathway network diagrams.

## 3 Results

### 3.1 Comparative carotenoid analysis in four Swiss chard cultivars

Carotenoids are pigments that confer yellow, orange, and red colours to plants and play vital roles in photosynthesis, protection against phototoxicity, pollinator attraction, and seed dispersal (Torres-Montilla & Rodriguez-Concepcion, 2021). Since the most visually apparent difference between the cultivars was the leaf petiole colour, with yellow and red in a couple of the cultivars, the contents of five carotenoids were investigated to determine whether carotenoids were responsible for the colour of the Swiss chard leaves (Table 1). The total carotenoids were highest in green and red cultivars and lowest in white. Lutein was the carotenoid that contributed the most to the total carotenoid content, and similar to the total carotenoid content, lutein was most abundant in the green and red cultivars and lowest in white. Zeaxanthin was the least abundant carotenoid and contributed the least to the total carotenoid content among the cultivars. Zeaxanthin was the most abundant in the red cultivar and the least abundant in the green cultivar, while its content in both the white and yellow cultivars was similar. The 13Z-β-carotene content was highest in the red cultivar, followed by the green cultivar, while the white and yellow cultivars had the lowest content. The second most abundant carotenoid of the total carotenoids was β-carotene, and it was least to most abundant in the order yellow, white, red, and green cultivars. The 9Z-β-carotene content was highest in the red and green cultivars, followed by the white and yellow cultivars. Overall, the cultivar exhibiting yellow and red colouration in the leaves was not significantly higher in carotenoid content than the cultivar with green leaves. These results were expected since previous research on the Swiss chard confirmed that the yellow and red pigments are mostly responsible for betalain rather than other pigments (Franzoni et al., 2024; Kugler, Stintzing, & Carle, 2004). Also, the nature of carotenoid localization in the cell and tissue implies most carotenoid contents may arise from the leaf blades (Lisiewska et al., 2006; Montoya-Arroyo et al., 2022; T. Sun et al., 2018; W. Sun et al., 2021). Together, these indicate that the yellow and red colours observed in the petioles of the two Swiss chard leaves may not be due to carotenoids.

**Table 1:** Primary and secondary metabolites were identified from the four different Swiss chard cultivars using GC-qMS and GC-TOFMS. Ratio/g represents a relatively quantified value using the peak area ratio to the internal standard (IS). A significant difference (*P* < 0.05) is labeled with different superscript letters (a, b, c, and d) based on the ANOVA followed by Tukey's HSD post hoc test. DW, dry weight.

	White	Green	Yellow	Red
*Carotenoid (μg/g DW)*				
Lutein	456.14 ± 19.24^b^	518.28 ± 19.56^a^	394.91 ± 20.52^c^	521.32 ± 10.50^a^
Zeaxanthin	3.52 ± 0.18^bc^	3.19 ± 0.23^c^	3.83 ± 0.32^b^	4.47 ± 0.22^a^
13Z-β-carotene	24.41 ± 2.07^c^	28.72 ± 2.00^b^	24.65 ± 1.74^c^	38.7 ± 1.02^a^
β-carotene	154.77 ± 5.20^b^	170.2 ± 14.18^a^	98.63 ± 5.04^c^	164.77 ± 7.35^ab^
9Z-β-carotene	17.31 ± 0.79^b^	19.04 ± 1.56^a^	8.06 ± 0.61^c^	19.83 ± 0.94^a^
Total carotenoid	656.15 ± 26.71^bc^	739.44 ± 33.85^ab^	530.07 ± 30.08^d^	749.08 ± 15.78^a^
*Betalain (μg/g DW)*				
Betacyanin	90.4 ± 10.74^c^	95.3 ± 15.66^c^	138.1 ± 7.63^b^	458.9 ± 12.21^a^
Betaxanthin	92.4 ± 5.88^c^	101.8 ± 14.98^c^	183.1 ± 5.19^b^	332.4 ± 10.02 ^a^
Total betalain	182.8 ± 16.07 ^c^	197.1 ± 30.63^c^	321.1 ± 12.23^b^	791.3 ± 22.23 ^a^
*Lipophilic (μg/g DW)*				
Eicosanol	11.08 ± 0.21^a^	11.13 ± 0.58^a^	11.66 ± 0.44^a^	11.01 ± 0.37^a^
Docosanol	14.07 ± 0.79^b^	11.36 ± 0.2^c^	12.87 ± 0.41^b^	16.22 ± 0.12^a^
Tetracosanol	37.78 ± 2.04^b^	31.45 ± 1.08^b^	32.01 ± 2.03^b^	46.66 ± 2.92^a^
Hexacosanol	349.54 ± 21.77^b^	221.54 ± 5.35^c^	276.94 ± 20.56^bc^	456.88 ± 34.37^a^
Heptacosanol	27.26 ± 2.2^a^	25.21 ± 6.6^a^	23.11 ± 3.42^a^	30.14 ± 1.51^a^
Octacosanol	2490.55 ± 350.52^ab^	1558.42 ± 324.29^b^	2416.18 ± 177.67^ab^	3002.49 ± 376.78^a^
α-Tocopherol	80.96 ± 9.74^a^	74.53 ± 2.45^a^	45.02 ± 3.44^b^	56.51 ± 0.90^b^
Triacontanol	486.99 ± 25.33^a^	524.28 ± 49.93^a^	397.64 ± 71.68^a^	549.86 ± 35.15^a^
Stigmasterol	75.02 ± 6.46^a^	81.04 ± 3.17^a^	84.44 ± 3.12^a^	89.13 ± 9.21^a^
β-Sitosterol	61.83 ± 2.47^ab^	65.67 ± 3.47^a^	51.43 ± 3.70^b^	63.53 ± 4.36^a^
Total lipophilic	3635.08 ± 495.49^ab^	2604.63 ± 343.44^b^	3351.31 ± 288.87^ab^	4322.43 ± 547.26^a^
*Phenolic acid (ratio/g)*				
Ferulic acid	1.30 ± 0.04^a^	0.61 ± 0.04^c^	1.18 ± 0.02^b^	0.49 ± 0.02^d^
Sinapinic acid	1.35 ± 0.15^a^	0.88 ± 0.12^b^	0.64 ± 0.03^b^	0.77 ± 0.05^b^
Total phenolic acid	2.65 ± 0.19^a^	1.49 ± 0.16^bc^	1.82 ± 0.05^b^	1.26 ± 0.07^c^
*Hydrophilic (ratio/g)*				
Pyruvic acid	3.02 ± 0.15^a^	1.87 ± 0.13^b^	0.85 ± 0.13^c^	1.19 ± 0.03^c^
Lactic acid	32 ± 2.07^b^	49.44 ± 9.6^ab^	65.47 ± 12.4^a^	44.64 ± 2.35^ab^
Alanine	36.3 ± 1.89^a^	35.68 ± 3.48^a^	18.78 ± 2.15^b^	36.29 ± 1.58^a^
Oxalic acid	61.26 ± 6^a^	54.39 ± 5.76^a^	58.46 ± 2.87^a^	61.15 ± 2.25^a^
Glycolic acid	0.71 ± 0.06^a^	0.8 ± 0.02^a^	0.38 ± 0.01^c^	0.5 ± 0.03^b^
Valine	2.24 ± 0.23^ab^	4.49 ± 1.61^a^	0.41 ± 0.12 ^b^	1.23 ± 0.19^b^
Serine	33.84 ± 1.23^a^	36.85 ± 1.58^a^	27.23 ± 1.22^b^	37.54 ± 0.25^a^
Glycerol	110.86 ± 3.97^b^	65.41 ± 1.5^c^	137.44 ± 2.5^a^	60.29 ± 0.47^c^
Phosphoric acid	437.96 ± 18.55^b^	519.38 ± 5.7^a^	257.52 ± 12.09^c^	405.89 ± 6.35^b^
Isoleucine	0.05 ± 0.00^a^	0.06 ± 0.01^a^	0.01 ± 0.00^b^	0.05 ± 0.00^a^
Proline	0.05 ± 0.00^b^	0.03 ± 0.00^c^	0.02 ± 0.00^d^	0.06 ± 0.00^a^
Glycine	19.25 ± 1.26^a^	22.63 ± 2.8^a^	17.1 ± 1.19^b^	24.44 ± 0.16^a^
Succinic acid	264.35 ± 7.82^a^	183.33 ± 3.31^b^	177.51 ± 1.85^b^	180.27 ± 1.89^b^
Glyceric acid	17.66 ± 0.4^a^	10 ± 0.24^d^	14.44 ± 0.59^b^	12.29 ± 0.22^c^
Fumaric acid	29.98 ± 0.46^a^	15.7 ± 0.43^b^	11.56 ± 0.58^c^	14.34 ± 0.45^b^
Threonine	0.04 ± 0.01^b^	1.78 ± 0.14^a^	1.83 ± 0.07^a^	0.13 ± 0.05^b^
β-Alanine	2.77 ± 0.08^c^	350.26 ± 7.63^a^	282.38 ± 7.19^b^	3.3 ± 0.04^c^
Malic acid	325.1 ± 13.91^a^	79.77 ± 10.19^c^	80.97 ± 4.88^c^	260.17 ± 2.99^b^
Aspartic acid	86.56 ± 5.79^d^	175.68 ± 8.29^b^	203.9 ± 3.77^a^	126.52 ± 1.92^c^
Pyroglutamic acid	163.98 ± 3.55^b^	22.43 ± 1.4^c^	14.11 ± 0.77^d^	222.36 ± 0.85^a^
GABA	38.95 ± 1.01^b^	57.38 ± 1.63^a^	37.17 ± 1.75^b^	22.94 ± 0.36^d^
Threonic acid	40.95 ± 0.86^b^	N.D.	N.D.	64.2 ± 0.6^a^
Cysteine	0.83 ± 0.06^a^	0.79 ± 0.1^a^	0.13 ± 0.01^b^	0.66 ± 0.07^a^
Glutamic acid	83.83 ± 5.2^c^	103.34 ± 4.15^b^	95.5 ± 3.95^b^	146.22 ± 0.88^a^
Phenylalanine	12.4 ± 0.33^b^	6.09 ± 0.4^c^	4.98 ± 0.27^d^	14.72 ± 0.23^a^
Xylose	16.73 ± 0.69^a^	11.42 ± 0.09^b^	6.86 ± 0.28^c^	7.83 ± 0.07^c^
Arabinose	31.83 ± 1.62^a^	11.31 ± 0.12^c^	16.18 ± 0.7^b^	5.79 ± 0.01^d^
Asparagine	2.87 ± 0.96^b^	2.54 ± 1.05^b^	1.5 ± 0.35^b^	6.68 ± 0.19^a^
Xylitol	2.62 ± 0.18^a^	1.89 ± 0.02^b^	2.45 ± 0.05^a^	2.76 ± 0.1^a^
Glutamine	37.97 ± 8.87^b^	38.16 ± 11.2^b^	13.43 ± 2.78^c^	64.59 ± 1.03^a^
Citric acid	152.13 ± 3.64^b^	167.02 ± 1.66^a^	151.02 ± 6.63^b^	163.72 ± 2.44^ab^
Quinic acid	0.43 ± 0.11^ab^	0.24 ± 0.02^b^	0.54 ± 0.08^a^	0.33 ± 0.01^ab^
Fructose	957.85 ± 22.65^a^	930.06 ± 21.15^a^	724.3 ± 29.87^b^	688.28 ± 18.35^b^
Mannose	6.39 ± 1.02^b^	13.62 ± 0.23^a^	12.02 ± 0.37^a^	6.12 ± 0.14^b^
Galactose	32.74 ± 0.82^b^	10.82 ± 0.14^c^	45.36 ± 1.51^a^	5.66 ± 0.14^d^
Glucose	361.12 ± 7.67^b^	407.24 ± 3.98^a^	320.46 ± 13.91^c^	269.57 ± 7.04^d^
Lysine	4.08 ± 0.22^a^	1.23 ± 0.04^bc^	0.93 ± 0.09^c^	1.59 ± 0.01^b^
Tyrosine	25.41 ± 0.39^d^	55.86 ± 1.02^b^	35.12 ± 1.49^c^	89.35 ± 0.86^a^
Inositol	66.5 ± 2.29^a^	30.03 ± 0.5^c^	39.13 ± 1.12^b^	35.33 ± 0.43^b^
Tryptophan	5.63 ± 0.57^b^	6.19 ± 0.48^b^	6.24 ± 0.43^b^	14.5 ± 0.2^a^
Fructose-6-phosphate	1.19 ± 0.06^c^	2.79 ± 0.05^a^	0.25 ± 0.03^d^	2.1 ± 0.05^b^
Glucose-6-phosphate	2.99 ± 0.28^c^	8.98 ± 0.49^a^	0.9 ± 0.04^d^	5.01 ± 0.17^b^
Sucrose	211.49 ± 7.44^c^	301.44 ± 6.53^a^	269.5 ± 10.58^b^	296.96 ± 1.61^a^
Raffinose	19.17 ± 0.4^a^	8.25 ± 0.47^b^	18.73 ± 0.91^a^	17.16 ± 0.91^a^
Total Hydrophilic	3744.06 ± 102.73^a^	3806.68 ± 44.24^a^	3173.06 ± 59.44^c^	3424.72 ± 34.47^b^

N.D. indicates Not detected.

### 3.2 Betalain content and gene analysis in four different Swiss chard cultivars

Since the carotenoid analysis was not consistent with the appearance of Swiss chard leaves, another group of compounds that may be responsible for the colour was investigated. Betalains comprise a group of pigments known to have antioxidant activity and are conserved only in some groups of plants in the Caryophyllales order (Brockington, Walker, Glover, Soltis, & Soltis, 2011; Nirmal et al., 2024). Previous studies have demonstrated that betalain in *B. vulgaris* is responsible for the leaf colouration (Bárta et al., 2020; Polturak et al., 2016; Pyo et al., 2004). Thus, the betalain content was analysed among the four cultivars to determine the pigments behind the colouration using a spectrophotometer (Table 1). White and green Swiss chard leaves were not significantly different in terms of betacyanin and betaxanthin contents. However, the yellow and red Swiss chard cultivar leaves had significantly higher betacyanin and betaxanthin contents than the white and green cultivar leaves, as was expected. The red cultivar showed the highest betacyanin and betaxanthin contents, followed by the yellow, green, and white cultivars. This indicates that betalain is present in all Swiss chard cultivars, and it is highest in the red cultivar, followed by the yellow cultivar.

However, the yellow cultivar exhibited a lower betaxanthin content than the red cultivar, which was unexpected since the red cultivar did not exhibit strong yellow pigments. Also, a previous study found that betaxanthin was more abundant in the yellow cultivar than in the red (Kugler et al., 2004). Thus, it was expected that the betalain biosynthesis pathway would differ at the genetic level between the cultivars. The expressions of genes encoding betalain synthesis enzymes that were identified in previous studies on beet (*B. vulgaris*) were investigated to understand the betalain pathway when comparing the different Swiss chard cultivars (**Figs. S2 and S3**) (Polturak et al., 2016). The expression of BvCYP76AD1, a key enzyme regulating overall betalain, was highest in the red cultivar, supporting that the red cultivar has the highest betacyanin and total betalain levels (**Fig. S3a and** Table 1). Unlike BvCYP76AD1, BvCYP76AD6 gene expression was not significantly different among the cultivars (**Fig. S3b**). BvDODA1 expression was significantly lower in the white and green cultivars than in the yellow and red cultivars, which was consistent with the overall betalain content (**Fig. S3c and** Table 1). The gene expression data were in agreement with the betalain content, indicating that the transcriptional regulation of betalain biosynthesis enzymes affecting the content difference between the four different Swiss chard cultivars is present.

### 3.3 Phenolic acid analysis in four different Swiss chard cultivars

Compounds other than the two pigments were further analysed to investigate the bioactive metabolites among the four cultivars. Phenylpropanoids are a group of secondary metabolites containing a benzene ring connected to a linear carbon chain (Hui et al., 2024). The compounds in phenylpropanoids are involved in plant stress responses, such as UV protection and response against pathogens, and are beneficial to human health as antioxidants (Lin & Eudes, 2020; Sharma et al., 2019; Yadav et al., 2020). Two phenolic acids in the phenylpropanoid subgroup (ferulic acid and sinapinic acid) were detected and quantified in the cultivars (Table 1). The ferulic acid content was highest in the white cultivar, followed by the yellow, green, and red cultivars. The sinapinic acid content was highest in the white cultivar, but there were no significant differences among the green, yellow, and red cultivars. Overall, the phenolic acid content was higher in the white cultivar than in the others and lowest in the red. These data indicate that part of the phenylpropanoid pathway is more active in the white cultivar and less active in the red cultivar.

### 3.4 Lipophilic compound analysis in four different Swiss chard cultivars

Lipophilic compounds in plants are among the key groups of compounds responsible for antioxidant activity (Szymanska, Latowski, Nowicka, & Strzałka, 2014). Thus, the contents of 10 lipophilic compounds were determined in the four cultivars. Policosanol is a group of lipophilic compounds that has recently been highlighted because of its benefits when consumed (Olatunji, Jimoh, Tukur, & Imam, 2022). In this analysis, seven compounds (eicosanol, docosanol, tetracosanol, hexacosanol, heptacosanol, octacosanol, and triacontanol) were considered policosanols (Table 1). Overall, the total lipophilic content was highest in the red cultivar, mainly due to the higher policosanol content. The pattern of policosanol content varied among the cultivars depending on the policosanol. The contents of eicosanol, heptacosanol, and triacontanol were not significantly different among the four cultivars. Docosanol had the highest content in the red cultivar, but it was similar in the yellow and red cultivars and lowest in the white cultivar. The red cultivar has a higher tetracosanol content than the other cultivars, with no significant differences among the other cultivars. Similarly, the red cultivar had a significantly higher hexacosanol content than the other cultivars, and the green cultivar had the lowest content. The content of octacosanol, a major active policosanol, was also highest in the red cultivar and lowest in the green cultivar. Phytosterols are another group of lipophilic compounds known to have strong antioxidant activity (Yoshida & Niki, 2003). Two phytosterols (stigmasterol and β-sitosterol) were comparatively analysed in the cultivars. The stigmasterol content was not significantly different among the cultivars. However, the β-sitosterol content was significantly lower in the yellow cultivar than in the other cultivars. α-Tocopherol, known as vitamin E, is an active lipophilic compound with antioxidant activity, and it was more abundant in the white and green cultivars than in the yellow and red cultivars (Tucker & Townsend, 2005). Lipophilic compound analysis showed that it was more abundant in the white and green cultivars than in the yellow and red cultivars. These results suggest that Swiss chard is a good source of policosanol and that the red cultivar is the best source of active policosanol among the cultivars.

### 3.5 In vitro antioxidant activity assay using four different Swiss chard cultivar extracts

Since differences in some individual phenolic compounds were observed among the cultivars, TPC and TFC were measured using the methanol extracts of the four cultivars (**Table S2**). TPC was highest in the yellow cultivar and lowest in the white cultivar, but was not significantly different in the green and red cultivars. Similarly, TFC was highest in the yellow cultivar and lowest in the white cultivar, while the red cultivar was statistically similar to the yellow cultivar, and the green cultivar was statistically similar to the white cultivar.

**Fig. 1:**
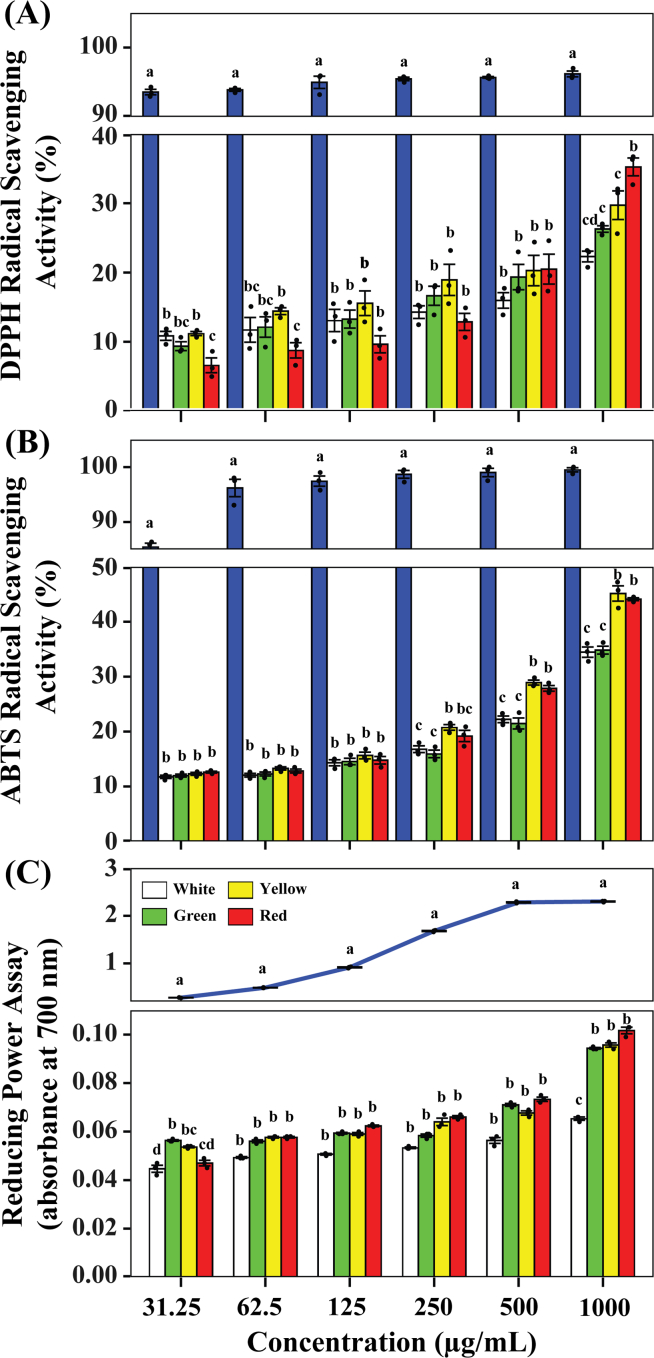
(A) DPPH presented as percent of inhibition, (B) ABTS presented as percent of inhibition, and (C) Reducing Power assay presented as absorbance at 700 nm in the four different Swiss chard extracts. The bar represents the mean, and the error bar represents the standard deviation (SD). A significant difference (*P* < 0.05) is labeled with different superscript letters (a, b, and c) based on the ANOVA followed by Tukey's HSD post hoc test.

An investigation of the metabolites and the TPC/TFC suggests that the antioxidant activity differs among the cultivars. Thus, the antioxidant activity was investigated in the extracts of the four cultivars. The antioxidant activity was confirmed with three assays: DPPH, ABTS, and RP. The red cultivar had the highest in antioxidant activity in DPPH at a concentration of 1000 μg/mL, while the trend was less obvious in the extract at a lower concentration (Fig. 1a). In the case of ABTS, both yellow and red exhibited significantly stronger antioxidants than the white and green cultivars at 1000 μg/mL, and the pattern was less obvious at a lower concentration (Fig. 1b). The RP assay also showed higher antioxidant activity in the red cultivar extract at a concentration of 1000 μg/mL compared to the white cultivar, but the results from this assay differed from those of the other assays, as the green cultivar exhibited a similar antioxidant effect as the red cultivar (Fig. 1c). Although the results varied between the assays, which may be due to differences in the chemical reaction and detection of antioxidant activity, the patterns observed throughout the assays were similar. Overall, the assay results were surprising because the antioxidant activity of the yellow cultivar did not reflect its high TPC and TFC. This indicates that there may be antioxidants in Swiss chard that are independent of TPC and TFC.

### 3.6 Metabolic profiling of identified metabolites in different Swiss chard cultivars

**Fig. 2:**
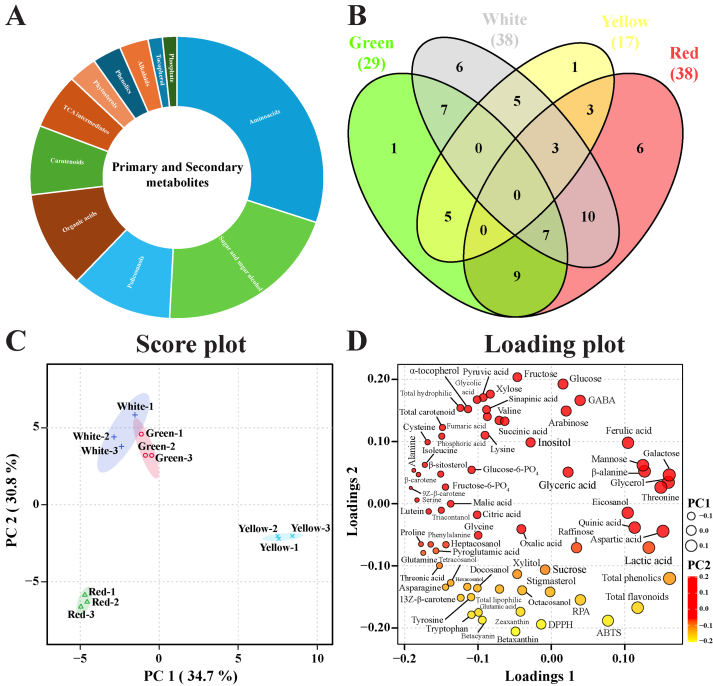
(A) Distribution of primary and secondary metabolites in different Swiss chard cultivars. (B) Venn diagram showing the distribution of seventy-one metabolites. Numbers shown outside the Venn diagram represent the total number of metabolites identified by the *Z*-score in each cultivar. (C) Score, and (D) loading plots of the PCA models of seventy-one differential metabolites identified in different Swiss cultivars. The circle represents the PC1, and the colour scale indicates the PC2 values of seventy-one metabolites.

Metabolic profiling analysis was performed to further identify antioxidants that were distinct from TPC/TFC and to understand the comprehensive relationship between the metabolites, cultivars, and antioxidant activity. A total of 63 metabolites (amino acids, sugars, sugar alcohols, policosanols, organic acids, carotenoids, citrate cycle (TCA cycle) intermediates, phytosterols, phenolics, alkaloids, ethanolamine, tocopherol, and phosphate) were identified from the different Swiss chard cultivars using HPLC, GC-TOF-MS and GC-qMS analysis (Fig. 2a **and** Table 1). *Z*-score analysis showed that the red and white cultivars consisted of 38 metabolites, whereas the green and yellow cultivars consisted of 29 and 17 metabolites, respectively. The relative abundance of metabolites, such as succinic acid, fumaric acid, arabinose, lysine, inositol, and sinapinic acid, was above average in the white cultivar compared to other cultivars, whereas the compounds, such as glutamic acid, betacyanin, asparagine, glutamine, tryptophan, and 13Z-β-carotene, showed above average abundance in the red cultivars compared to other cultivars based on the Z-score (Fig. 2b). Gamma-aminobutyric acid (GABA) and eicosanol showed above average abundance in the green and yellow cultivars, respectively, compared to the other cultivars.

PCA was performed to identify the metabolite differences among the four Swiss chard cultivars. The red and yellow cultivars were well separated from each other, whereas the green and white cultivars were not separated. The PCA score plot showed a 34.7% (principal component 1, PC1) and 30.8% (PC2) variance with two-component analysis (Fig. 2c). In PC1, the main metabolites that contributed to differential separation were galactose, glycerol, aspartic acid, threonine, lactic acid, β-alanine, mannose, quinic acid, eicosanol, and ferulic acid which showed positive eigenvector values of 0.15979, 0.1587, 0.15294, 0.14941, 0.13366, 0.12701, 0.12468, 0.11341, 0.10416, and 0.10302, respectively, and 9*Z*-β-carotene, alanine, serine, β-carotene, proline, glutamine, isoleucine, cysteine, lutein, and phenylalanine had negative eigenvector values of −0.19158, −0.18786, −0.18287, −0.18134, −0.17717, −0.17373, −0.17273, −0.16948, −0.16671, and − 0.16417, respectively (Fig. 2d **and Table S3**). Based on the *Z*-score and PC1, eicosanol was identified as the main metabolite separating the yellow cultivar from the other cultivars. Regarding the PC2 loading metabolites, the top 10 metabolites, including fructose, glucose, xylose, pyruvic acid, GABA, glycolic acid, α-tocopherol, sinapinic acid, arabinose, and fumaric acid, showed a positive value; the compounds that showed negative values were as follows: betaxanthin, betacyanin, tryptophan, glutamic acid, zeaxanthin, 13Z-β-carotene, tyrosine, stigmasterol, octacosanol, and docosanol (Fig. 2d **and Table S3**). Z-score and PC2 analysis together showed that these 4 compounds (glutamic acid, betacyanin, tryptophan, and 13Z-β-carotene) mainly separated the red cultivar from the other cultivars. However, since the white and green cultivars were not separated in the PCA model, PLS-DA was performed.

**Fig. 3:**
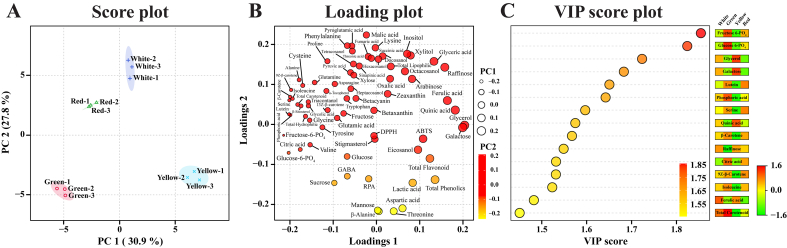
(A) Score and (B) loading plots of the PLS-DA models of seventy-one differential metabolites identified in different Swiss chard cultivars. The circle represents the PC1, and the colour scale indicates the PC2 values of seventy-one metabolites. (C) Important compounds that differentiate Swiss chard cultivars, as determined by VIP scores from the PLS-DA model. On the right, each colored square represents the relative concentrations of metabolites identified across different Swiss chard cultivars. The green and red colours show the lowest to highest values, respectively. The top 15 important compounds corresponding to different Swiss chard cultivars are shown in descending order based on the VIP score. Inside the box, the red-to-yellow scale shows high to low values based on the VIP score. (For interpretation of the references to colour in this figure legend, the reader is referred to the web version of this article.)

PLS-DA showed a clear separation among the cultivars, showing 30.9 and 27.8% of the variance in PC1 and PC2, respectively (Fig. 3a). This distinct separation may have been due to glycerol, galactose, quinic acid, ferulic acid, raffinose, glyceric acid, eicosanol, lactic acid, arabinose, and xylitol, which showed positive eigenvector values of 0.20366, 0.19939, 0.18219, 0.16394, 0.15814, 0.13304, 0.095997, 0.083745, 0.082279, and 0.079159, respectively, and fructose-6-phosphate, glucose-6-phosphate, lutein, serine, β-carotene, phosphoric acid, 9Z-β-carotene, isoleucine, alanine, and citric acid, which had eigenvector values of −0.21461, −0.20303, −0.20181, −0.20126, −0.19952, −0.19917, −0.19724, −0.19397, −0.17883, and − 0.17619, respectively, in PC1 (Fig. 3b **and Table S4**). Regarding the PC2 loading metabolites, the top 10 metabolites, namely malic acid, phenylalanine, pyroglutamic acid, lysine, threonic acid, inositol, xylitol, succinic acid, fumaric acid, and hexacosanol, showed a positive value, and the compounds with a negative value were as follows: β-alanine, threonine, mannose, aspartic acid, sucrose, lactic acid, GABA, glucose, glucose-6-phosphate, and eicosanol, (Fig. 3b **and Table S4**). Based on Z-score analysis, six main compounds were abundant in the white cultivar, and the Z-score with PC2 analysis together showed that four of these compounds (succinic acid, fumaric acid, lysine, and inositol) separated the white cultivar from the other cultivars. However, we were not able to identify the main metabolites that differentiated the green cultivar from the other cultivars using the PLS-DA model. Therefore, the identification of further metabolites that are distinct in the white cultivar is necessary. VIP score plot analysis showed that metabolites, such as fructose-6-phosphate, glucose-6-phosphate, glycerol, galactose, lutein, phosphoric acid, serine, quinic acid, β-carotene, raffinose, citric acid, 9Z-β-carotene, isoleucine, ferulic acid, and total carotenoid, had significant VIP values (> 1) (Fig. 3c). PCA is an unsupervised method; hence, it explains these patterns only descriptively. PLS-DA with [m] components yielded with a maximum of 3 components to search, a 5-fold cross-validation (CV) method, the Q^2^ performance measure, and a label-permutation test (*n* = 1000). The models with 1, 2, and 3 components achieved the following results: R^2^ (0.96336, 0.99495, and 0.99661), Q^2^ (0.8966, 0.98276, and 0.9811), and CV accuracy (0.13333, 0.63333, and 1.0%), respectively. The cross-validation analysis of the PLS-DA model showed that a positive Q^2^ reproduces predictability and non-overfitting. The permutation test indicated a performance above chance (empirical *p* = 0.008) **(Fig. S4 and S5)**. This suggests that the accumulation patterns of primary and secondary metabolites differ among Swiss chard cultivars.

**Fig. 4:**
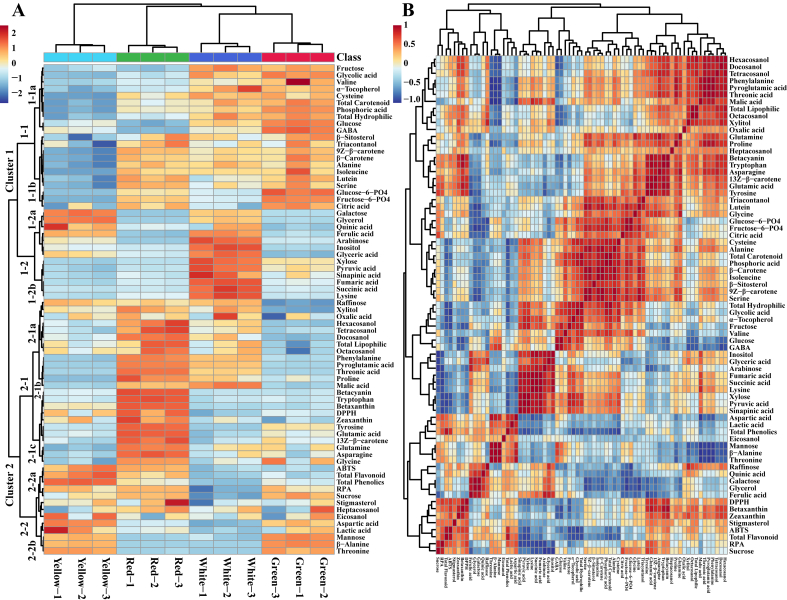
(A) Heatmap showing variations in the content of seventy-one metabolites identified in different Swiss chard cultivars. The low and high metabolites are represented in blue and red, respectively. (B) Pearson's correlation coefficient analysis of seventy-one identified metabolites from different Swiss chard cultivars. The correlation matrix for sets of compounds is represented as colored boxes; the correlation coefficient is shown as blue (low) or red (high), as displayed in the colour guide. (For interpretation of the references to colour in this figure legend, the reader is referred to the web version of this article.)

The heat map showed that most metabolites were significantly more abundant in the red cultivar, followed by the white, green, and yellow cultivars. The heat map was separated into two main clusters: clusters 1 and 2 (Figs. 4a **and S6**). Clade 1 was further split into four clades, namely 1–1 and 1–2, and cluster 2 was classified into clusters 2–1 and 2–2. Clade 2–1 was again subdivided into three clades, namely 2-1a, 2-2b, and 2-1c, and cluster 2–2 was subdivided into two clusters, namely 2-2a and 2-2b. Cluster 1 comprised metabolites that were abundant in the white, green, and red cultivars, whereas only a few metabolites were abundant in the yellow cultivar. Clade 1–1 consisted of compounds that were significantly abundant in the green cultivar, followed by the white and red cultivars. Subcluster 1-1a formed a separate group with the metabolites abundant in the green cultivar, followed by the white and red cultivars, whereas in this subcluster, none of the metabolites showed the highest abundance in the yellow cultivar. Similarly, the yellow cultivar did not show significant accumulation of the compounds in subcluster 1-1b; however, in this cluster, the compounds were considerably more abundant in the green and red cultivars, followed by the white cultivar. Cluster 1–2 consisted of compounds that were significantly more abundant in the white cultivar, and only a few metabolites were most abundant in the yellow and green cultivars. In subcluster 1-2a, the metabolites were only rich in the white and yellow cultivars, whereas in subcluster 1-2b, the metabolites were considerably more abundant in the white cultivar. Cluster 2 was mainly divided based on the compounds that were considerably more abundant in the red cultivar. Clade 2–1 formed a cluster of metabolites abundant in the red, yellow, and green cultivars. Subcluster 2-1a consisted of abundant metabolites in red, followed by white cultivars, whereas subcluster 2-1b was divided based on the metabolites abundant in the red cultivar. In subcluster 2-1c, the compounds rich in the red cultivar and the few slightly more abundant in the white and green cultivars formed a separate cluster. Subcluster 2-2a was composed of metabolites that were more abundant in both red and yellow cultivars, whereas subcluster 2-2b formed a separate cluster in which the compounds were rich in the yellow and green cultivars.

**Fig. 5:**
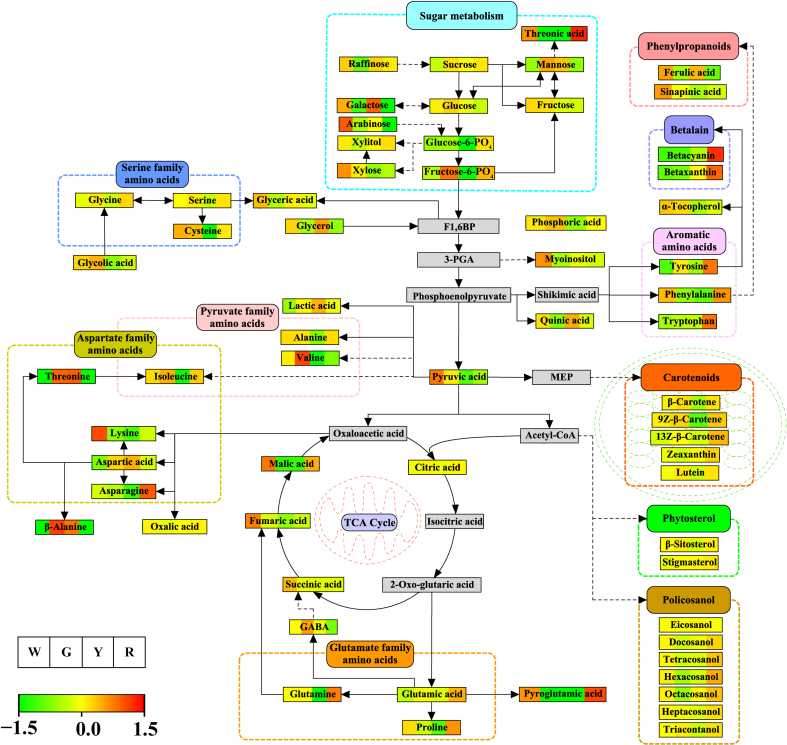
A diagrammatic representation of metabolite pathways comparing identified metabolites among Swiss chard cultivars. The intensity of the green to red colour shows the unit variance scaling data, as shown in the colour guide. W- White, G – Green, Y – Yellow, and R – Red. (For interpretation of the references to colour in this figure legend, the reader is referred to the web version of this article.)

Specifically, the TCA cycle intermediate products, such as succinic acid, fumaric acid, glyceric acid, malic acid, and pyruvic acid, were considerably higher in the white cultivar, whereas citric acid was abundant in both the green and red cultivars (Fig. 5). Most sugars and sugar alcohols showed distinct accumulation among cultivars. The sugars and sugar alcohols, such as glucose, fructose, and xylose, showed abundant accumulation in the green and white cultivars, whereas galactose, arabinose, glycerol, and inositol had high contents in the yellow and white cultivars. In addition, the mannose content was considerably high in the green and yellow cultivars. The raffinose and xylitol contents were significantly high in the red, yellow, and white cultivars. Similarly, sucrose was abundant in the green, red, and yellow cultivars. Individual amino acids, such as alanine, cysteine, isoleucine, serine, and valine, were abundant in the green, red, and white cultivars. The aspartic acid, β-alanine, and threonine contents were significantly high in the green and yellow cultivars, whereas the proline content was highest in the red and white cultivars. The asparagine, glutamine, glycine, and tyrosine contents were significantly abundant in the red cultivar, followed by the green cultivar. The tryptophan and lysine contents were highest in the red and white cultivars, respectively. Similarly, phenylalanine, the shikimate pathway derivative, was abundant in the red and white cultivars. Interestingly, 13Z-β-carotene and zeaxanthin were more abundant in the red cultivar than in the other cultivars, whereas carotenoids, such as 9Z-β-carotene, β-carotene, and lutein, were significantly more abundant in the green, red, and white cultivars (Fig. 5). The organic acids also showed differential accumulation among the cultivars. The pyruvic acid and glycolic acid contents were high in the green and white cultivars, whereas the white and red cultivars had high threonic acid and oxalic acid contents. Lactic acid was higher in the green and yellow cultivars than in the other cultivars. Quinic acid and glyceric acid were abundant in the white cultivars. Betacyanin and betaxanthin were remarkably more abundant in the red cultivar than in the other cultivars. The β-sitosterol content was high in the green, red, and white cultivars. The policosanol contents (hexacosanol, tetracosanol, docosanol, and octacosanol) were significantly higher in the red and white cultivars, but the heptacosanol and eicosanol contents were similar in all cultivars. The heat map showed that the red and white cultivars are more abundant in overall metabolites than the green and yellow cultivars. Similarly, the red and white cultivars showed the highest accumulation of total lipophilic compounds, whereas the total hydrophilic compounds were highest in the green and white cultivars.

The correlations between the identified metabolites among the Swiss chard cultivars were examined using Pearson's correlation (Fig. 4b). Phenylalanine is an important amino acid, and it acts as a precursor phenylpropanoid and flavonoid biosynthesis. Phenylalanine showed a positive correlation with sinapinic acid; however, it showed a negative correlation with ferulic acid. Regarding flavonoids, both betacyanin and betaxanthin showed a positive correlation with phenylalanine. Similarly, phenylalanine showed a positive correlation with all TCA cycle intermediates (citric acid, fumaric acid, succinic acid, and malic acid). TCA cycle intermediates are also important precursors for the biosynthesis of several amino acids. Except for lysine, the TCA cycle intermediate citric acid showed a positive correlation with all amino acids. In addition, citric acid also had an indirect link to carotenoid biosynthesis, showing a positive correlation with all identified carotenoid compounds, such as 13Z-β-carotene, 9Z-β-carotene, β-carotene, lutein, zeaxanthin, and total carotenoid. Citrate also plays a vital intermediary role in supplying acetyl-CoA for fatty acid biosynthesis and is used for phytosterol and fatty acid biosynthesis. Citric acid showed a positive correlation with the identified phytosterol metabolites (β-sitosterol and stigmasterol). Similarly, this citrate showed a positive correlation with a few policosanols (heptacosanol, triacontanol, and tetracosanol), whereas it showed a negative correlation with docosanol, eicosanol, hexacosanol, and octacosanol. Sucrose acts as a signalling molecule that triggers the key genes in betalain and carotenoid biosynthesis. This finding was consistent with the correlation analysis, which showed that sucrose had a positive correlation with all identified betalain and carotenoid compounds.

Pearson's correlation between metabolites and antioxidant assays identified several additional non-phenolic and non-flavonoid antioxidant candidates (Fig. 4b). Carotenoids, such as 13Z-β-carotene and zeaxanthin, were positively correlated with antioxidant assays and were more abundant in the red cultivar than in the yellow cultivar, even though they are neither a phenolic nor a flavonoid **(**Fig. 4b **and** Table 1**)**. This could explain the higher antioxidant activity in the red cultivar than in the yellow cultivar, independent of TPC/TFC (Figs. 1 **and Table S2**). Similarly, lipophilic compounds that are not phenolic or flavonoids, such as docosanol, tetracosanol, hexacosanol, heptacosanol, octacosanol, and triacontanol, known as policosanol, were higher in the red cultivar than in the other cultivars, and most of them showed a positive correlation with the antioxidant assays (Fig. 4b **and** Table 1). Some hydrophilic metabolites showed a positive correlation with the antioxidant assays and were not phenolic or flavonoid antioxidants, such as glutamic acid, glutamine, and pyroglutamic acid, which were higher in the red cultivar than in the other cultivars (Fig. 4b **and** Table 1). This demonstrates that the red cultivar generally had a stronger and more stable antioxidant activity than the other cultivars, not only because of phenolics and flavonoids but also because of metabolites that are not in the category of phenolic and flavonoids.

**Fig. 6:**
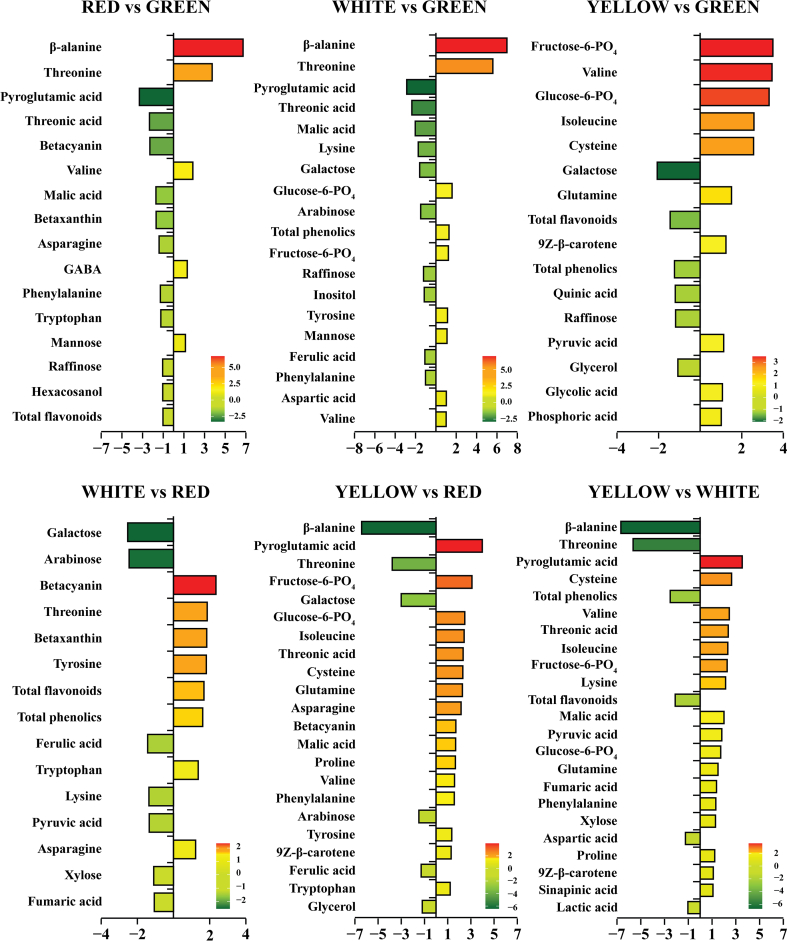
Differential metabolites annotated based on the log-2 fold change value between different Swiss chard cultivars. Fold changes were calculated by dividing the appropriate cultivar (green/red, green/white, green/yellow, red/white, red/yellow, white/yellow). The significance of the difference was analysed by *t*-test (*p* < 0.05). (For interpretation of the references to colour in this figure legend, the reader is referred to the web version of this article.)

More comparisons were made based on the identified metabolites to understand the impacted pathways and the additional metabolite differences among the cultivars. HPLC, GC-TOFMS and GC-qMS showed that 16, 19, 16, 15, 22, and 23 differentially expressed metabolites were identified based on the log-2 fold change value between red vs green, white vs green, yellow vs green, white vs red, yellow vs red, and yellow vs white cultivars, respectively (Fig. 6). By comparing metabolites among cultivars, the results showed that the majority of the metabolites were significantly more abundant in the red cultivar. The betacyanin and betaxanthin contents were only significantly high in the red cultivar. The red cultivar had the highest betacyanin and betaxanthin contents, which were 4.89- and 3.23-fold higher than the green cultivar and 5.07- and 3.57-fold higher than the white cultivar. In addition, betacyanin and betaxanthin showed a positive correlation with DPPH, ABTS, and RP assays (Fig. 4b). This indicates that betacyanin and betaxanthin might play crucial roles in the antioxidant activity. The betacyanin content was higher in the red cultivar than in the yellow cultivar, showing a 3.26-fold higher content. In addition, the highest TFC and TPC were found in the red cultivar. The 9Z-β-carotene and pyroglutamic acid contents were highest in the red cultivar. Based on the overall results, most of the metabolites were high in the red cultivar compared to the other cultivars. The white cultivar was the second most abundant in metabolites, followed by the green and yellow cultivars. Betacyanin and betaxanthin were most abundant in the red cultivar, followed by the yellow cultivar. These results show that the red cultivar might have the highest antioxidant activity compared to the other cultivars due to betalain and other non-flavonoids, such as 9Z-β-carotene and pyroglutamic acid.

**Fig. 7:**
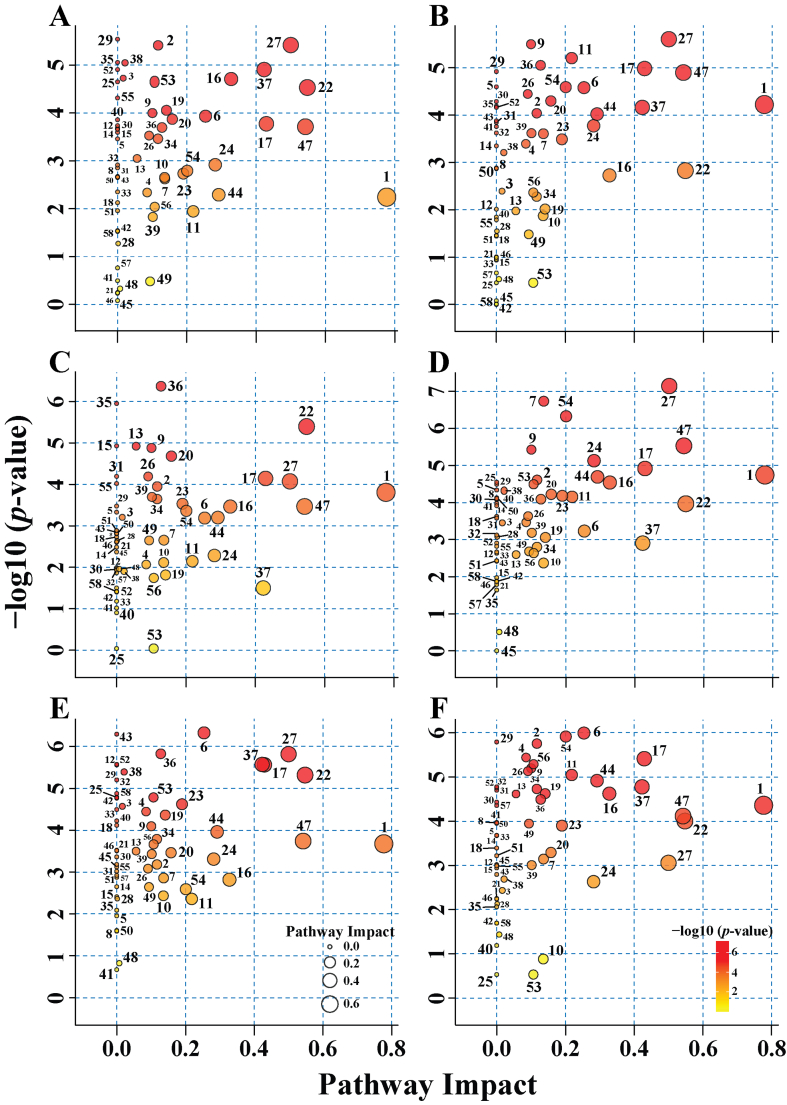
KEGG pathway of differentially expressed metabolites between different Swiss chard cultivars (t-test, *p* < 0.05). (A) Green vs Red, (B) Green vs White, (C) Green vs Yellow, (D) Red vs White, (E) Red vs Yellow, (F) White vs Yellow. Significantly changed pathways are specified based on enrichment and topology analyses. Colour intensity (yellow to red) represents increasing statistical significance, and variation in the size of the circle represents the pathway impact. 1) Alanine, aspartate and glutamate metabolism, 2) Amino sugar and nucleotide sugar metabolism, 3) Arginine and proline metabolism, 4) Arginine biosynthesis, 5) Ascorbate and aldarate metabolism, 6) β-alanine metabolism, 7) Butanoate metabolism, 8) C5-Branched dibasic acid metabolism, 9) Carbon fixation in photosynthetic organisms, 10) Carotenoid biosynthesis, 11) Citrate cycle (TCA cycle), 12) Cyanoamino acid metabolism, 13) Cysteine and methionine metabolism, 14) D-amino acid metabolism, 15) Fatty acid degradation 16) Fructose and mannose metabolism, 17) Galactose metabolism, 18) Glucosinolate biosynthesis, 19) Glutathione metabolism, 20) Glycerolipid metabolism, 21) Glycerophospholipid metabolism, 22) Glycine, serine and threonine metabolism, 23) Glycolysis / Gluconeogenesis, 24) Glyoxylate and dicarboxylate metabolism, 25) Indole alkaloid biosynthesis, 26) Inositol phosphate metabolism, 27) Isoquinoline alkaloid biosynthesis, 28) Lipoic acid metabolism, 29) Lysine biosynthesis, 30) Lysine degradation, 31) Monobactam biosynthesis, 32) Nicotinate and nicotinamide metabolism, 33) Nitrogen metabolism, 34) Pantothenate and CoA biosynthesis, 35) Pentose and glucuronate interconversions, 36) Pentose phosphate pathway, 37) Phenylalanine metabolism, 38) Phenylalanine, tyrosine and tryptophan biosynthesis, 39) Phenylpropanoid biosynthesis, 40) Porphyrin metabolism, 41) Propanoate metabolism, 42) Purine metabolism, 43) Pyrimidine metabolism, 44) Pyruvate metabolism, 45) Selenocompound metabolism, 46) Sphingolipid metabolism, 47) Starch and sucrose metabolism, 48) Steroid biosynthesis, 49) Sulfur metabolism, 50) Terpenoid backbone biosynthesis, 51) Thiamine metabolism, 52) Tropane, piperidine and pyridine alkaloid biosynthesis, 53) Tryptophan metabolism, 54) Tyrosine metabolism, 55) Ubiquinone and other terpenoid-quinone biosynthesis, 56) Valine, leucine and isoleucine biosynthesis, 57) Valine, leucine and isoleucine degradation, and 58) Vitamin B6 metabolism. (For interpretation of the references to colour in this figure legend, the reader is referred to the web version of this article.)

Fig. 7 shows the identified differentially expressed metabolites mapped to the *A. thaliana* KEGG pathways. From the comparison of the Swiss chard cultivars, the top five metabolic pathways that were considerably impacted based on the −log *p*-value and pathway impact scores were alanine, aspartate, and glutamate metabolism, glycine, serine, and threonine metabolism, starch and sucrose metabolism, isoquinoline alkaloid biosynthesis, and galactose metabolism.

## 4 Discussion

Previous nutrient analysis has confirmed that Swiss chard is rich in carotenoids, which are the well-known yellow and red pigments in plants. However, most previous studies have focused on the total carotenoids or a small number of carotenoid compounds in a single Swiss chard cultivar (Ivanović et al., 2019; Mzoughi et al., 2019; Reif, Arrigoni, Schärer, Nyström, & Hurrell, 2013). In this study, several carotenoids were investigated among four Swiss chard cultivars that differ in their leaf petiole colours, and the total carotenoids were lowest in the yellow cultivar, indicating that the yellow and red leaf petiole colours may not be relevant to carotenoids (Table 1). Previous studies comparatively analysed the carotenoids between the leaf blades and the petiole in stinging nettle and dill, found that carotenoids are also more abundant in leaf blades than in petioles, indicating carotenoids may be less likely to be responsible for the pigments behind the petiole (Lisiewska et al., 2006; Montoya-Arroyo et al., 2022). This may be due to the specific localisation of the carotenoids in the plant cells. Carotenoids are known to be located in the chloroplast and to assist photosynthesis (T. Sun et al., 2018). Thus, tissues with more chloroplasts are naturally richer in carotenoids, and it has previously been confirmed that leaf blades have more chloroplasts than petioles (W. Sun et al., 2021). This indicates that most of the carotenoid content in this study originated from the leaf blade, but not the petiole, supporting the carotenoid content results. Although the carotenoid contents did not represent the petiole colour, there were some differences in the carotenoid content among the cultivars (Table 1). However, a study analysing the carotenoid pathway among Swiss chard cultivars is still absent. Thus, future studies on metabolomics and genetics are needed to investigate the mechanism behind the carotenoids depending on the Swiss chard cultivars.

Betalain analysis showed that all cultivars contained betalain. Specifically, the red cultivar had the highest content of both betacyanin and betaxanthin, and the white cultivar had the lowest content **(**Table 1**)**. This result was consistent with a previous study that investigated the betalin contents in several *B. vulgaris* cultivars, in which the red beet exhibited a higher betalain content than yellow and white beetroot (Bárta et al., 2020). In contrast, another study compared the betalain contents among Swiss chard cultivars and found that the orange–yellow cultivar that resembled the red cultivar in this study was less abundant in the overall betalain content than the yellow cultivar (Kugler et al., 2004). The study found that the yellow cultivar had lower betaxanthin content than the orange–yellow cultivar, while the yellow cultivar only had a small amount of betacyanin that was not quantifiable. This may be due to genetic differences between the yellow cultivars that were used. Previously, research investigating the function of genes regarding betalain biosynthesis found that transient expression of *BvDODA1* with *BvCYP76AD1* in tobacco increased betacyanin production, while the betaxanthin level was increased when *BvDODA1* was expressed with *BvCYP76AD6* in tobacco (Polturak et al., 2016). This indicates that betacyanin and betaxanthin production could be significantly altered depending on the betalain biosynthetic gene expression pattern in each cultivar. The highest expression of the *BvCYP76AD1* cultivar explains the higher betalain content, including betaxanthin, in the red cultivar compared to the others used in this study. In detail, strong expression of *BvCYP76AD1*, which synthesises L-3,4-dihydroxyphenylalanine (L-DOPA) that is eventually converted into both betacyanin and betaxanthin, with strong expression of *BvDODA1*, might have resulted in higher betacyanin and betaxanthin in the red cultivar than in the other cultivars in this study (**Figs. S2, S3, and** Table 1). In contrast, the expression of *BvCYP76AD1* and *BvDODA1* in the orange–yellow cultivar used by Kugler et al. (2004) may not be strong enough to exceed the overall betalain content in the yellow cultivar. Similarly, *BvCYP76AD1*, which is an essential enzyme to produce betacyanin, may not be expressed well in the yellow cultivar used by Kugler et al. (2004), while other genes essential for betaxanthins, such as *BvCYP76AD6* and *BvDODA1*, are expressed. In this study, gene expression patterns were consistent with the overall betalain content of the Swiss chard cultivars (**Fig. S3 and** Table 1). Together, these show that yellow and red pigments in Swiss chard are responsible for betalain synthesis, and the production of the pigments may vary depending on the expression of biosynthetic genes in each cultivar. However, biosynthetic pathway enzymes could also be regulated by post-translational modification, which could modify the enzyme activity and the abundance of the enzyme in the plants. Thus, further study is needed to investigate the post-translational modification of the enzymes, which could partially modulate the betalain contents along with the gene expression, to advance understanding of betalain biosynthesis.

Phenolic acid analysis identified two compounds: ferulic acid and sinapinic acid (Table 1). Phenolic acid was especially abundant in the white cultivar compared to the other cultivars. Similar to our study, previous research comparing the phenolic acid content between the red and white Swiss chard cultivars found that ferulic acid was more abundant in the leaves of the white cultivar than in the red cultivar (Pyo et al., 2004). Other studies have identified phenylpropanoids, such as vitexin, caffeic acid, *p*-Coumaric acid, myricitrin acid, and rosmarinic acid, which were not identified in this study (Gennari et al., 2011; Moyo et al., 2018; Mzoughi et al., 2019; Wessler et al., 2025). However, the amount of the compounds that were quantified in each study varied, possibly due to differences in extraction and quantification methods. In addition, these differences between the studies may be the result of the cultivars and harvest conditions, as observed previously in other plants (Gök, Özdüven, & Açıkgöz, 2024; Van Binh et al., 2024). Together, these variations in the study and the lack of phenylpropanoid investigations in Swiss chard cultivars highlight the necessity of additional studies on phenylpropanoid production in Swiss chard in connection with various factors, such as cultivars and stressors affecting production.

Previously, some research has examined lipophilic compounds, including tocophenol, fatty acids, and a small portion of policosanol, in Swiss chard (Almeida et al., 2025). This study detected eicosanol, docosanol, tricosanol, and tetracosanol in Swiss chard leaves. Several policosanols, including eicosanol, docosanol, and tetracosanol, were quantified in this study (Table 1). In addition, more policosanols, such as hexacosanol, heptacosanol, octacosanol, and triacosanol, that were not confirmed in previous studies, have been detected in this study (Table 1). Identification of additional policosanols from Swiss chard may be very significant, not only because it has not been previously confirmed, but also because octacosanol, known to be the main active policosanol, accumulated remarkably in Swiss chard (Table 1) (Harrabi, Boukhchina, Mayer, & Kallel, 2009). Other lipophilics, such as α-tocopherol and β-sitosterol, were detected in this study, which is consistent with previous studies on *B. vulgaris* (Almeida et al., 2025; Schmidt, Kuhnt, & Adam, 1994; Schmitt, Gaspar, & Hagège, 1994). This result suggests that policosanol, especially octacosanol, is abundant in Swiss chard overall, which may have biological effects.

KEGG pathway analysis, further conducted to investigate the pathway impact based on the identified metabolites, showed that alanine, aspartate, and glutamate metabolism were significantly impacted. These pathways play an important role in plant nitrogen metabolism and stress response (Diab & Limami, 2016; O'Leary & Plaxton, 2015; Yoneyama & Suzuki, 2020; Zayed et al., 2023). These amino acids act as a central intermediate in the integration, storage, and reallocation of nitrogen within the plant (Yoneyama & Suzuki, 2020). Alanine mainly accumulates under hypoxic or stress conditions, such as drought and flooding, and helps to sustain the cellular redox balance and energy homeostasis (Agrawal, Chunletia, Badigannavar, & Mondal, 2024). Glutamates act as a primary amino group donor in the synthesis of other amino acids through transamination reactions, which are directly linked to carbon and nitrogen metabolism (Forde & Lea, 2007). Several studies have also found that glutamate treatment induces antioxidant metabolites, such as flavonoids and vitamin C in blueberries, and phenolic acids, including caffeic acid and chlorogenic acid, in cut carrots (Pérez-León et al., 2023; Zhang et al., 2024). This suggests that glutamate may enhance the stress resistance of plants by improving their bioactive metabolites. Aspartate is also a precursor for some essential amino acids, such as lysine, methionine, and threonine, which will participate in the asparagine synthesis, an important nitrogen transport and storage molecule (O'Leary & Plaxton, 2015). Additionally, aspartate could increase tolerance of salt stress in wheat and tomato by promoting antioxidants such as TPC, TFC, ascorbic acid, anthocyanin, and glutathione (Akladious & Abbas, 2013; Sadak et al., 2022). Hence, alteration in the pathway might lead to shifts in nitrogen assimilation efficiency, interconversion of amino acids, and adaptive responses to environmental stress, which reflect the changes in the plant's overall metabolic status and resilience mechanisms. From the overall report, it is shown that alanine, aspartate, and glutamate metabolism play a crucial role in the stress-responsive mechanism in Swiss chard cultivars. However, due to the non-availability of Swiss chard-specific KEGG library, compounds or metabolic pathways distinctive to Swiss chard, as well as unknown or poorly annotated features, may be collapsed into distributed KEGG nodes. Hence, pathway impact values may undervalue the impact of species-specific or highly focused metabolic pathways.

The DPPH radical scavenging assay showed that the extracts from the red cultivar had the strongest antioxidant activity, although the TPC in the yellow cultivar extract was highest. However, in the case of the ABTS and RP assays, the extracts from the red and yellow cultivars had similar antioxidant activities, and the antioxidant activity in the green cultivar was similar to that in the red cultivar in the RP assay (Fig. 1). This variation among assays might have been caused by the differences in the mechanism and antioxidant efficiency of compounds depending on the assay used to measure the antioxidant activity (Abramovič, Grobin, Poklar Ulrih, & Cigić, 2018; Chaves, Santiago, & Alías, 2020). This indicates that the antioxidant activity of some groups of compounds may not appear depending on the antioxidant assay. In addition, the yellow cultivar had a higher TPC than the red cultivar, indicating that the yellow cultivar may have a stronger antioxidant activity, which was not the case (Fig. 1 **and Table S2**). TPC and the distinct tendencies of different antioxidant assays suggest the existence of non-phenolic and non-flavonoid antioxidants (**Table S2**). The antioxidant activity may be higher in the red cultivar than in the yellow cultivar in the DPPH assay due to metabolites with strong scavenging activity that cannot be detected in TPC and TFC quantification because they are neither phenolic compounds nor flavonoids (Fig. 1a **and Table S2**).

The comprehensive analysis showed that metabolites in the carotenoids, policosanol, and glutamic acid derivatives may have contributed to the antioxidant activity in the red cultivar (Fig. 4b). Carotenoids are known to have a strong antioxidant effect by efficiently removing singlet oxygen through direct interaction, even though they are neither phenolic nor flavonoid (Stahl & Sies, 2003). The carotenoid characteristic partially explains the strong positive correlation observed between the antioxidant assay and carotenoids that were more abundant in the red cultivar than in the yellow cultivar in this study (Fig. 4b **and** Table 1). Policosanols from plants have shown antioxidant activity by directly inhibiting the oxidation of lipoproteins induced by reactive oxygen species (ROS) (Cho, Kim, Komatsu, & Uehara, 2023; Cho, Kim, Nam, Kang, & Baek, 2023; Menéndez et al., 2000). A previous study identified some of the policosanols in Swiss chard; however, it did not note policosanols as a significant antioxidant candidate in Swiss chard, as the study lacked comparative metabolomic analysis between different Swiss chard cultivars (Almeida et al., 2025). In this study, policosanols and antioxidant activity showed a positive correlation; a similar result was obtained in milk thistle, which showed a positive correlation between antioxidant activity and policosanols. Moreover, in this study, we identified several policosanols compounds which have not been previously reported (Harrabi, Ferchichi, Bacheli, & Fellah, 2018) (Fig. 4a **and** Table 1). From this result, it is expected that policosanol plays a significant role in antioxidant activity in Swiss chard. Some glutamic acid derivatives, such as glutamine and pyroglutamic acid, which were abundant in the red cultivar, have been reported to contribute to antioxidant activity by previous studies (Table 1). In detail, glutamine provides antioxidant activity as a precursor of glutathione, a strong antioxidant, and by participating as a key precursor in proline biosynthesis, which removes ROS via redox balance modulation(Amores-Sánchez & Medina, 1999; Rejeb, Abdelly, & Savouré, 2014). Also, a previous study found that glutamine supplementation increased antioxidant activity in humans (Nemati, Alipanah-Moghadam, Molazadeh, & Naghizadeh Baghi, 2019), indicating that glutamine has strong antioxidant activity in vivo. In the case of the pyroglutamic acid, it was found to increase the antioxidant activity of pyroglutamic acid-treated lettuce extract and exhibit strong antioxidant activity in vitro assays, indicating the possibility of pyroglutamic acid removing ROS directly, although the direct mechanism behind this is still unclear (Bilska et al., 2018; Jiménez-Arias et al., 2019). Although some studies have shown that glutamic acid derivatives exhibit antioxidant activity, the potential of glutamic acid derivatives as antioxidants has not received much attention in Swiss chard. Thus, this finding suggests glutamic acid derivatives present in the Swiss chard may play a significant role in antioxidant activity. An additional analysis of the metabolites between the cultivars with the antioxidant assay showed that betalains are strong antioxidants in Swiss chard, which is consistent with a previous study (Belhadj Slimen, Najar, & Abderrabba, 2017). In summary, the results show that metabolites such as carotenoids, policosanols, and glutamic acid derivatives, which were not previously recognized to have antioxidant activity in Swiss chard, showed strong positive correlations with antioxidant assays suggesting the metabolites as potential antioxidants. However, further study needs to experimentally demonstrate the antioxidant activity of carotenoids, policosanols, and glutamic acid derivatives in Swiss chard.

Overall, the results indicate that TPC and TFC are not always directly proportional to the antioxidant activity, meaning that TPC and TFC may not fully explain the antioxidant activity. However, by comparatively analysing the antioxidant activity, TPC, TFC, and non-phenolic/non-flavonoid metabolites together in Swiss chard, it was possible to identify antioxidants (policosanols and glutamic acid derivatives) that did not receive attention previously. Thus, this study has identified potential antioxidants in Swiss chard cultivars, where breeding was traditionally conducted by selecting desirable traits, and continuous breeding could lead to a distinct genetic background in the bred population. Thus, it is expected that the cultivar-dependent modulation of metabolites observed in this study may be due to genetic differences between the cultivars. A previous study reported that Swiss chard has the highest genetic diversity among the group within *B. vulgaris* (Galewski & McGrath, 2020). However, the current investigation into comparative genetic/genomic analysis within Swiss chard cultivars is insufficient. Further comparative genome-wide analysis in Swiss chard cultivars may provide more clarified and profound knowledge not only on betalain biosynthesis but also on the regulation of other valuable metabolites.

## 5 Conclusion

In this study, the composition of compounds and antioxidant activity in the four Swiss chard cultivars were investigated using a metabolomic approach. This study discovered several non-flavonoid metabolites (policosanols, carotenoids, etc.) that may be responsible for the antioxidant activity and were not previously highlighted in Swiss chard. These metabolites might not have been revealed if the antioxidant activity had only been investigated with TPC and TFC, with only a few compounds relevant to these contents. Thus, this study has emphasised the importance of analysing antioxidant activity with metabolomic profiling rather than studying it with a small group of secondary metabolites. Also, the metabolomic profiles revealed in the study suggest that Swiss chard, especially the red cultivar, is a good source of policosanol, indicating that Swiss chard could be used to make dietary supplements and consumed as a health-promoting vegetable. The metabolic profiles further suggest that Swiss chard breeding using the yellow and red cultivars could maximize both antioxidant activity and nutritional value. However, this study was not able to confirm which metabolites were responsible for the phenolic and flavonoid components contributing to the high TPC and TFC in the yellow Swiss chard cultivar. Thus, further studies involving phenolics and flavonoid component analysis in connection with Swiss chard cultivars are necessary to uncover unknown antioxidants in Swiss chard. In addition, further molecular studies investigating gene regulation behind the newly discovered antioxidant metabolites in Swiss chard in this study will help understand the regulation of metabolites.

## CRediT authorship contribution statement

**Chanung Park:** Writing – original draft, Software, Methodology, Formal analysis, Data curation. **Jae Kwang Kim:** Software, Resources, Methodology, Formal analysis, Data curation. **Jinsu Lim:** Software, Methodology, Formal analysis, Data curation. **Kihyun Kim:** Software, Methodology, Formal analysis, Data curation. **Haejin Kwon:** Software, Methodology, Formal analysis, Data curation. **Eun Sol Cho:** Software, Methodology, Formal analysis, Data curation. **Ye Jin Kim:** Software, Methodology, Formal analysis, Data curation. **Moon-Sub Lee:** Software, Methodology, Formal analysis, Data curation. **Sujatha Ramasamy:** Software, Methodology, Formal analysis, Data curation. **Ramaraj Sathasivam:** Writing – review & editing, Writing – original draft, Project administration, Methodology, Conceptualization. **Sang Un Park:** Writing – review & editing, Supervision, Resources, Project administration, Funding acquisition, Conceptualization.

## Funding

This work was supported by BK21 FOUR Program by 10.13039/501100002462Chungnam National University Research Grant, 2025.

## Declaration of competing interest

The authors declare that they have no known competing financial interests or personal relationships that could have appeared to influence the work reported in this paper.

## Data Availability

Data will be made available on request.

## Appendix A Supplementary data

Supplementary material Image 1
