# Supplementary material for: Comprehensive metabolomics and phytochemical analyses identified important metabolites involved in the antioxidant activity of four Swiss chard cultivars (Beta vulgaris L. var. cicla) with different leaf colours
Source: Food Chem X. 2026 Jan 25;34:103587. doi: 10.1016/j.fochx.2026.103587 (PMC12887366; doi:10.1016/j.fochx.2026.103587)
Supplement: Supplementary file 1 — Supplementary material [file mmc1.docx]

**Supplementary Materials**

**Comprehensive metabolomics and phytochemical analyses identified important metabolites involved in the antioxidant activity of four Swiss chard cultivars (*Beta vulgaris* L. var. cicla) with different leaf colours**

**Chanung Park^a,†^, Jae Kwang Kim^b,†^, Jinsu Lim^a^, Kihyun Kim^a^, Haejin Kwon^c^, Eun Sol Cho^b^, Ye Jin Kim^b^, Moon-Sub Lee^d^, Sujatha Ramasamy^e^, Ramaraj Sathasivam^a,f,*^, Sang Un Park^a,c,*^**

^a^ Department of Crop Science, Chungnam National University, 99 Daehak-ro, Yuseong-gu, Daejeon 34134, Republic of Korea; [chanungpark92@gmail.com](mailto:chanungpark92@gmail.com) (C.P.); [jshs6762@naver.com](mailto:jshs6762@naver.com) (J.L.); [rlarlgus1130@naver.com](mailto:rlarlgus1130@naver.com) (K.K.); [kwonhaejin42@o.cnu.ac.kr](mailto:kwonhaejin42@o.cnu.ac.kr) (H.K.)

^b^ Division of Life Sciences and Convergence Research Center for Insect Vectors, Incheon National University, Yeonsu-gu, Incheon 22012, Republic of Korea; [kjkpj@inu.ac.kr](mailto:kjkpj@inu.ac.kr) (J.K.K.); [202202502@inu.ac.kr](mailto:202202502@inu.ac.kr) (E.S.C.); [201721047@inu.ac.kr](mailto:201721047@inu.ac.kr) (Y.J.K.)

^c^ Department of Smart Agriculture Systems, Chungnam National University, 99 Daehak-ro, Yuseong-gu, Daejeon 34134, Republic of Korea

^d^ Department of Crop Science, Chungbuk National University, 1 Chungdae-ro, Seowon-gu, Cheongju, 28644, Republic of Korea; [mlee128@chungbuk.ac.kr](mailto:mlee128@chungbuk.ac.kr) (M.-S.L.)

^e^ Institute of Biological Sciences, Faculty of Science, University of Malaya, 50603, Kuala Lumpur, Malaysia; [sujatha@um.edu.my](mailto:sujatha@um.edu.my) (S.R.)

^f^ Department of Integrative Agriculture, College of Agriculture and Veterinary Medicine, United Arab Emirates University, P.O. Box 15551, Al Ain, Abu Dhabi, United Arab Emirates.

**^†^**Chanung Park and Jae Kwang Kim contributed equally to this work.

___________________________________________________________________________

*Corresponding authors:

E-mail: [ramarajbiotech@gmail.com](mailto:ramarajbiotech@gmail.com) (R.Sathasivam); [supark@cnu.ac.kr](mailto:supark@cnu.ac.kr) (S.U. Park)


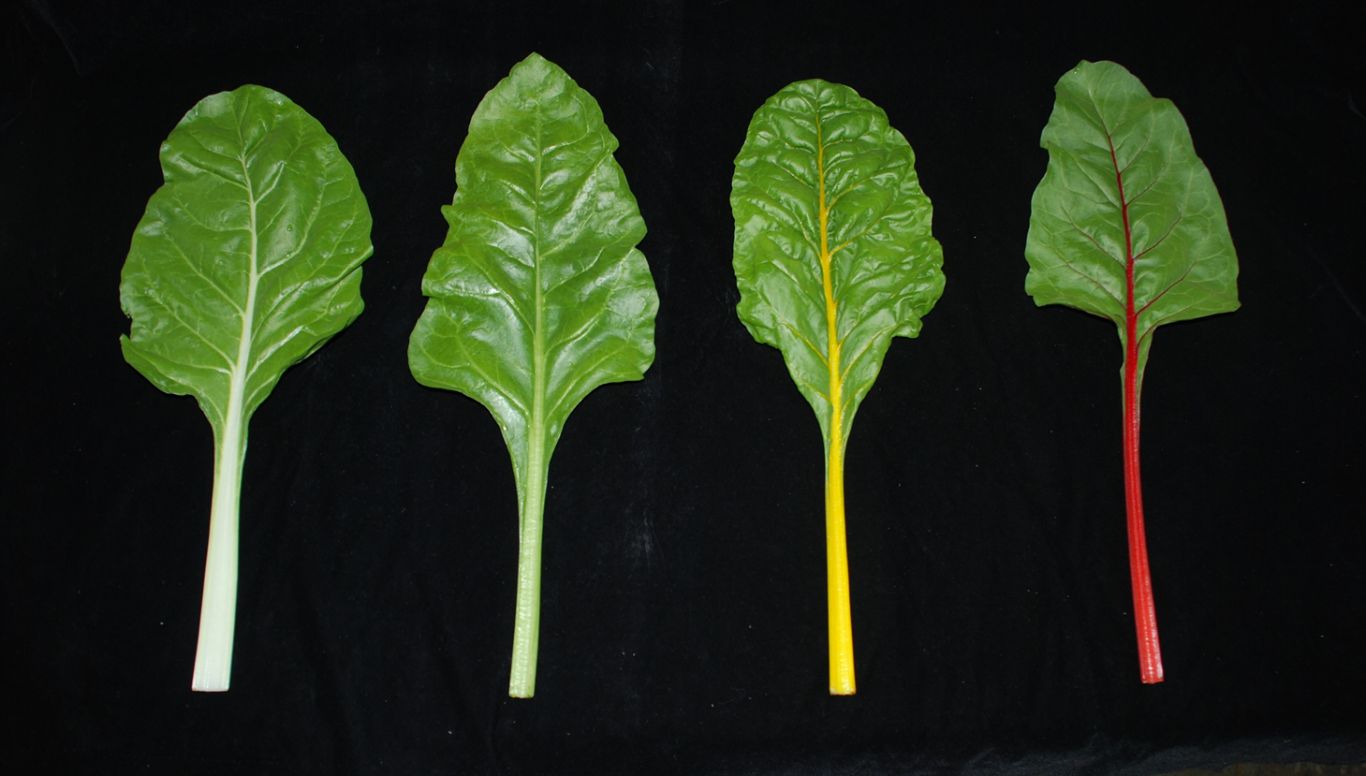


Fig. S1. Leaves of swiss chard (*Beta vulgaris* L. var. cicla). ‘Back Gyeong’ (white), ‘Cheong Gyeong’ (green), ‘Hwang Gyeong’ (yellow), and ‘Ruby Red’ (red) from left to right.


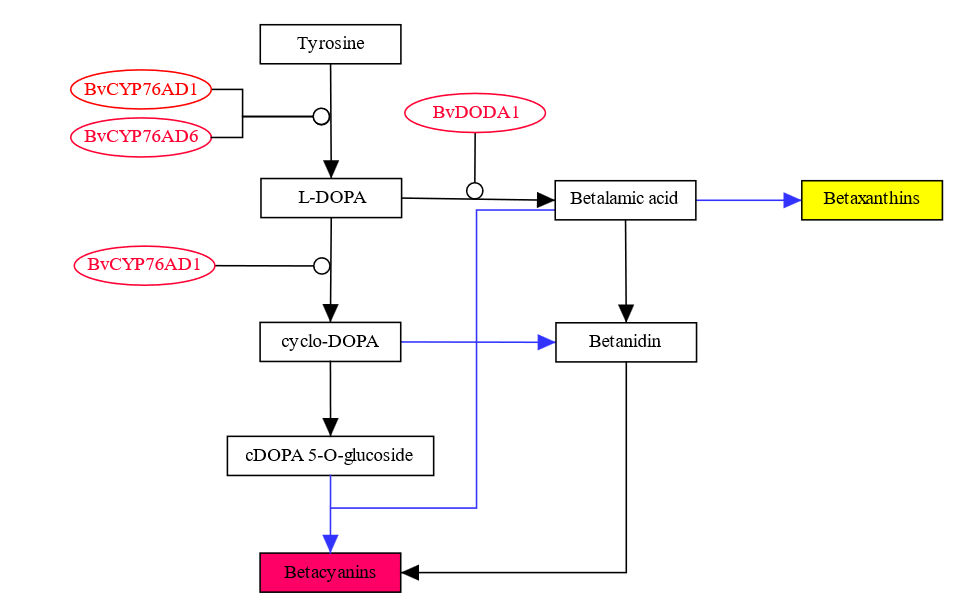


Fig. S2. Betalain biosynthesis pathway in *B. vulgaris*. BvCYP76AD1 and BvCYP76AD6 catalyze the reaction, converting tyrosine to L-DOPA. BvCYP76AD1 also converts L-DOPA to cyclo-DOPA, which is a key compound to produce precursors of betacyanin. BvDODA1 converts L-DOPA to betalamic acid, another key compound for betacyanin synthesis and the precursor of betaxanthin at the same time that is spontaneously converted into betaxanthin. Black rectangles represent metabolites, and red ovals represent enzymes catalyzing the reaction. The blue line indicates a spontaneous reaction.


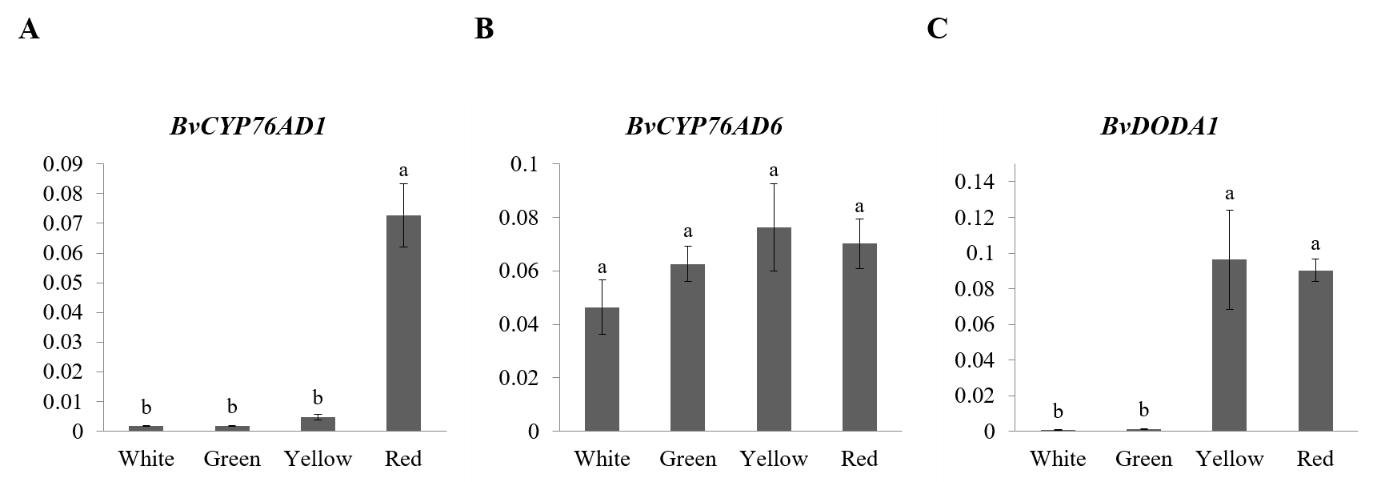


Fig. S3. Gene expression of betalain biosynthetic gene relative to BvActin in the four different Swiss chard cultivars. (A) *BvCYP76AD1*, (B) *BvCYP76AD6*, (C) *BvDODA1*. A significant difference (*P* < 0.05) is labeled with different superscript letters (a and b) based on the ANOVA followed by Tukey’s HSD post hoc test.


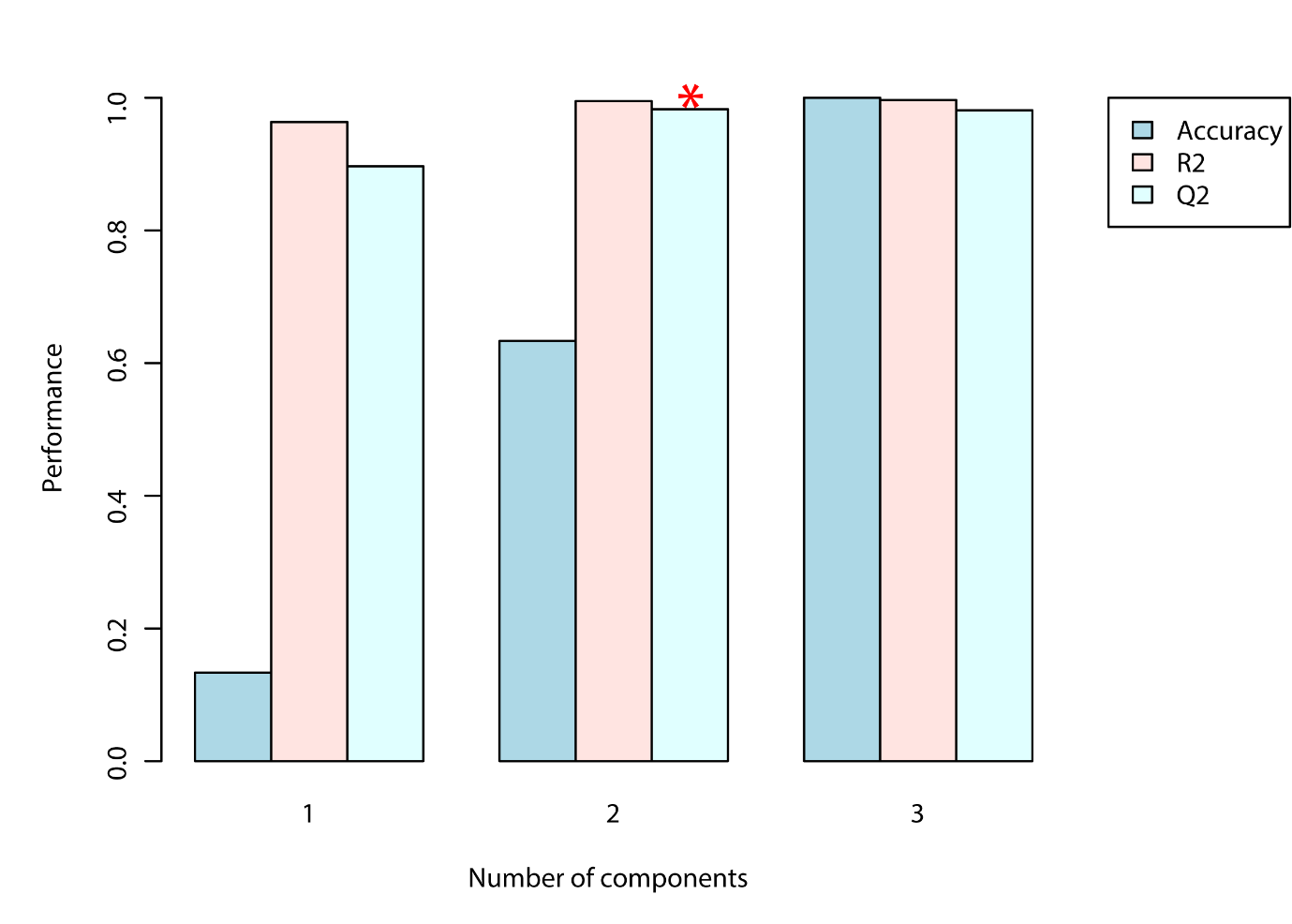


**Fig. S4.** Cross-validation of the PLS-DA with a positive Q2 reproduces predictability and non-overfitting of the model.


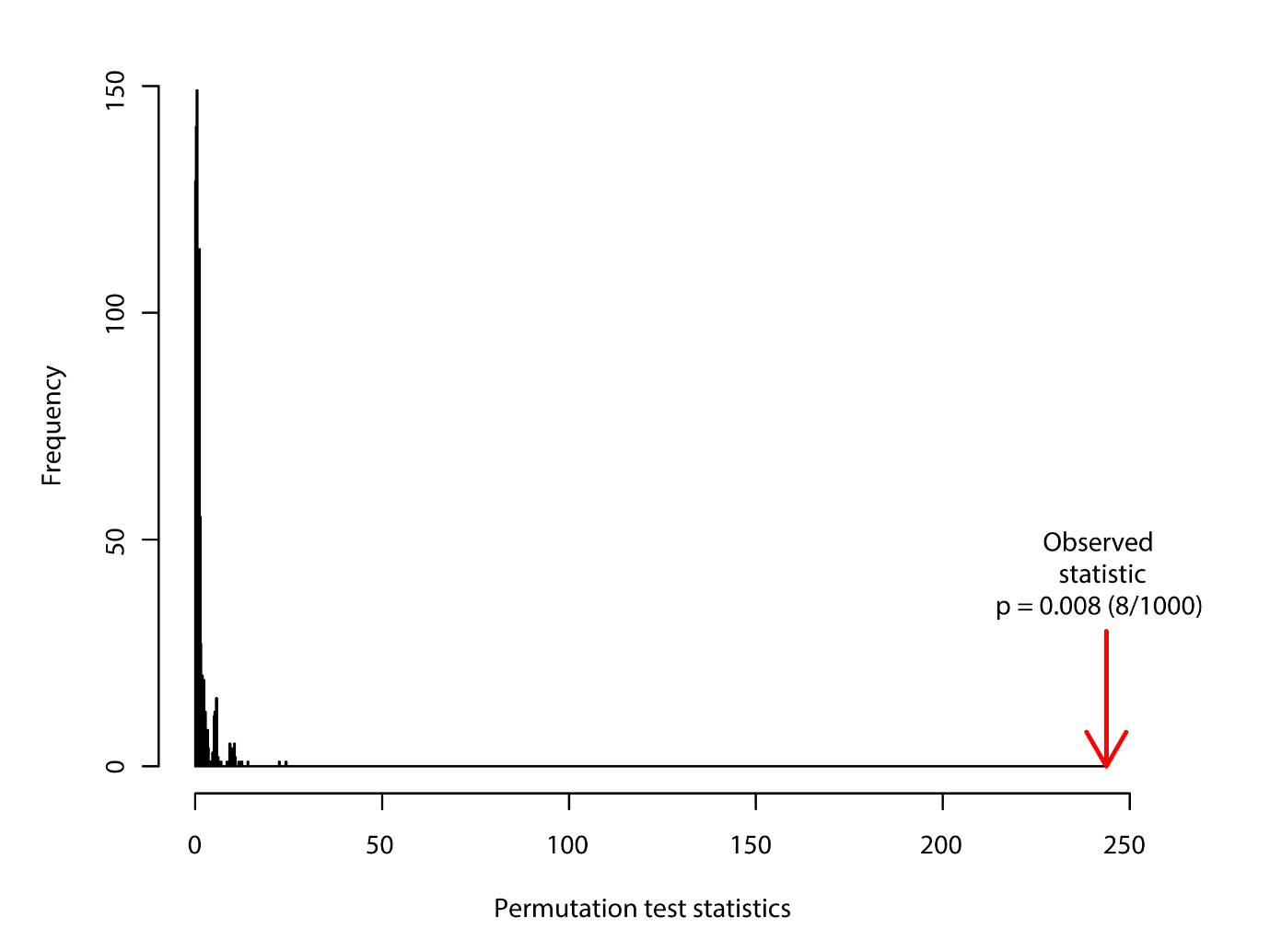


**Fig. S5.** PLS-DA label-permutation test (n=1000); observed statistic (*p* =0.008). The histogram shows the null distribution of the chosen performance statistic computed under cross-validation with cultivar labels randomly permuted (n = 1000 permutations). The red arrow marks the statistic obtained for the model fit to the true (unpermuted) labels. The empirical one-sided p-value equals the proportion of permuted statistics ≥ the observed value (8/1,000), giving *p* = 0.008. The permutation test used the same cross-validation scheme and model settings as the fitted PLS-DA.


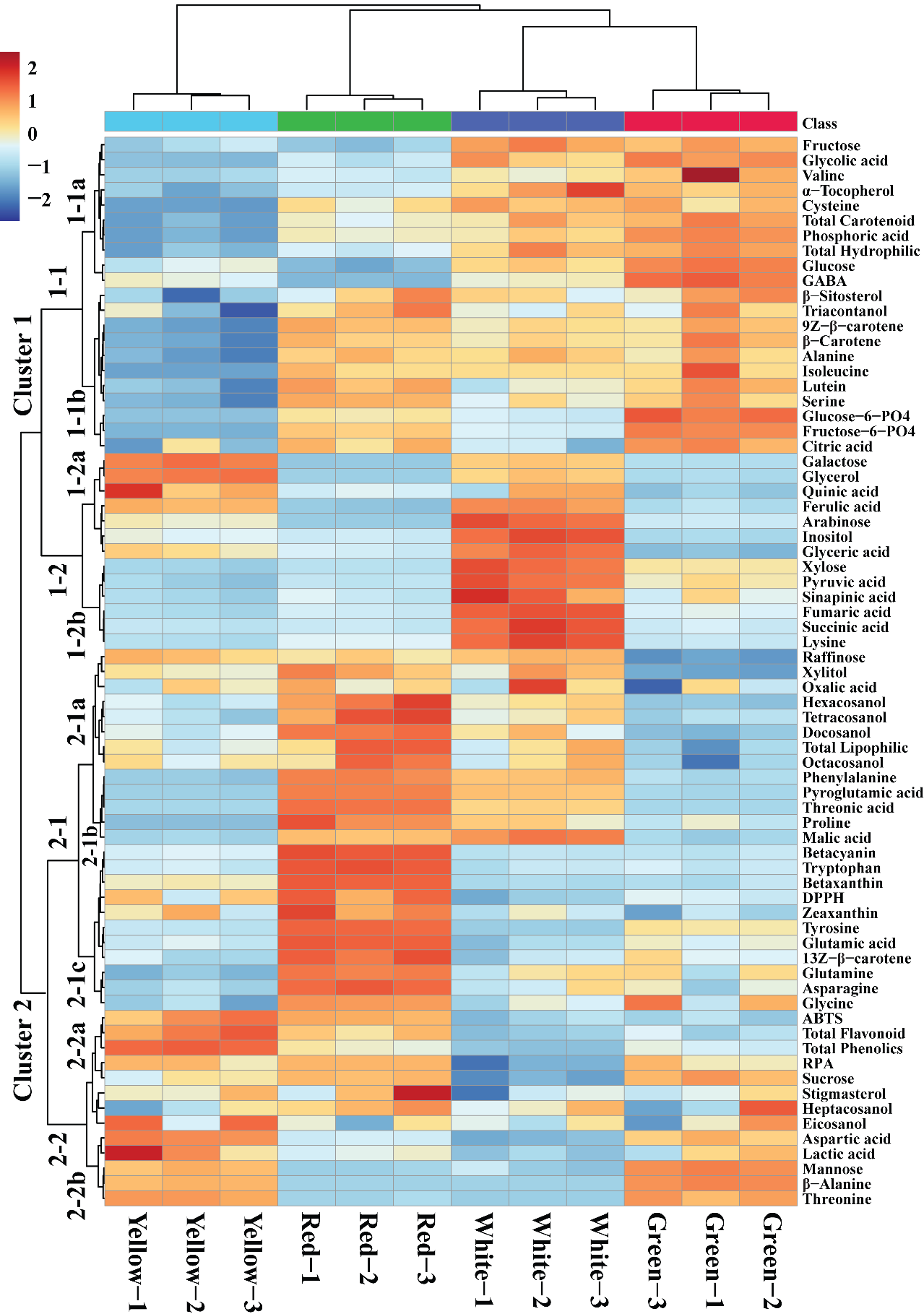


**Fig. S6** Heatmap showing variations in the content of seventy-one metabolites identified in different Swiss chard cultivars. The metabolites that are low and high are represented in blue and red, respectively.

**Table S1.** List of primer used for the qRT-PCR analysis.

| **Gene** | **Primer** | **Sequence**  (5’ to 3’) |
| --- | --- | --- |
| BvACT | BvACT-qRT-F | tctatccttgcatctctcag |
| BvACT | BvACT-qRT-R | atcatactcgcccttggaga |
| BvCYP76AD1 | BvCYP76AD1-qRT-F | cttttcagtggaattagcccacc |
| BvCYP76AD1 | BvCYP76AD1-qRT-R | cccaatatcttccataatgttcca |
| BvCYP76AD6 | BvCYP76AD6-qRT-F | cttcatctcgtaggcttcctcc |
| BvCYP76AD6 | BvCYP76AD6-qRT-R | gatgaggcttttcgccaaga |
| BvDODA1 | BvDODA1-qRT-F | cattggttcaggaagtgcaa |
| BvDODA1 | BvDODA1-qRT-R | acgaagccatgaatcaaagg |

**Table S2.** Total phenolic contents (TPC) and flavonoid contents (TFC) were calculated based on the gallic acid equivalent (GAE) and quercetin equivalent (QE) from four different *Beta vulgaris* L. leaves using a spectrophotometer. A significant difference (*P* < 0.05) is labeled with different superscript letters (a, b, and c) based on the ANOVA followed by Tukey's HSD *post hoc* test. DW, dry weight.

|  | **White** | **Green** | **Yellow** | **Red** |
| --- | --- | --- | --- | --- |
| Total Phenolic content (mg GAE/g DW) | 0.37 ± 0.04^c^ | 0.91 ± 0.12^b^ | 2.14±0.04^a^ | 1.14±0.12^b^ |
| Total Flavonoid content (mg QE/g DW) | 0.69 ± 0.13^b^ | 1.09 ± 0.35^b^ | 2.97 ± 0.35^a^ | 2.22 ± 0.28^a^ |

**Table S3.** Twenty key metabolites based on the PCA with loading scores.

| **PC1** | | **PC2** | |
| --- | --- | --- | --- |
| **Metabolites** | **Loadings** | **Metabolites** | **Loadings** |
| Galactose | 0.15979 | Fructose | 0.20336 |
| Glycerol | 0.1587 | Glucose | 0.19283 |
| Aspartic acid | 0.15294 | Xylose | 0.17565 |
| Threonine | 0.14941 | Pyruvic acid | 0.16999 |
| Lactic acid | 0.13366 | GABA | 0.16674 |
| β-Alanine | 0.12701 | Glycolic acid | 0.16652 |
| Mannose | 0.12468 | α-tocopherol | 0.15121 |
| Quinic acid | 0.11341 | Sinapinic acid | 0.15077 |
| Eicosanol | 0.10416 | Arabinose | 0.14958 |
| Ferulic acid | 0.10302 | Fumaric acid | 0.13945 |
| 9Z-β-carotene | -0.19158 | Betaxanthin | -0.20622 |
| Alanine | -0.18786 | Betacyanin | -0.18838 |
| Serine | -0.18287 | Tryptophan | -0.17991 |
| β -carotene | -0.18134 | Glutamic acid | -0.17608 |
| Proline | -0.17717 | Zeaxanthin | -0.17428 |
| Glutamine | -0.17373 | 13Z-β-carotene | -0.15277 |
| Isoleucine | -0.17273 | Tyrosine | -0.15163 |
| Cysteine | -0.16948 | Stigmasterol | -0.14203 |
| Lutein | -0.16671 | Octacosanol | -0.13973 |
| Phenylalanine | -0.16417 | Docosanol | -0.13695 |

**Table S4.** Twenty key metabolites based on the PLS-DA with loading scores.

| **PC1** | | **PC2** | |
| --- | --- | --- | --- |
| **Metabolites** | **Loadings** | **Metabolites** | **Loadings** |
| Glycerol | 0.20366 | Malic acid | 0.22341 |
| Galactose | 0.19939 | Phenylalanine | 0.19642 |
| Quinic acid | 0.18219 | Pyroglutamic acid | 0.19565 |
| Ferulic acid | 0.16394 | Lysine | 0.19197 |
| Raffinose | 0.15814 | Threonic acid | 0.18887 |
| Glyceric acid | 0.13304 | Inositol | 0.17685 |
| Eicosanol | 0.095997 | Xylitol | 0.17176 |
| Lactic acid | 0.083745 | Succinic acid | 0.17127 |
| Arabinose | 0.082279 | Fumaric acid | 0.17009 |
| Xylitol | 0.079159 | Hexacosanol | 0.16469 |
| Fructose-6-phosphate | -0.21461 | β-alanine | -0.21679 |
| Glucose-6-phosphate | -0.20303 | Threonine | -0.2166 |
| Lutein | -0.20181 | Mannose | -0.21417 |
| Serine | -0.20126 | Aspartic acid | -0.20965 |
| β-carotene | -0.19952 | Sucrose | -0.14656 |
| Phosphoric acid | -0.19917 | Lactic acid | -0.14611 |
| 9Z-β-carotene | -0.19724 | GABA | -0.12857 |
| Isoleucine | -0.19397 | Glucose | -0.07908 |
| Alanine | -0.17883 | Glucose-6-phosphate | -0.07075 |
| Citric acid | -0.17619 | Eicosanol | -0.06321 |

**Table S5.** Details of PLS-DA cross-validation of four Swiss chard cultivars.

| **Measure** | **1 Compenents** | **2 Components** | **3 Components** |
| --- | --- | --- | --- |
| Accuracy | 0.13333 | 0.63333 | 1.0 |
| R2 | 0.96336 | 0.99495 | 0.99661 |
| Q2 | 0.8966 | 0.98276 | 0.9811 |
